# Colorectal neoplasia-specific amino acid profiles and their diagnostic potential: a systematic review

**DOI:** 10.1007/s12094-026-04278-9

**Published:** 2026-03-28

**Authors:** Roza C. M. Opperman, Sofie Bosch, Eduard A. Struys, Awa Hassan, Faridi S. Jamaludin, Tim G. J. de Meij, Evelien Dekker, Nanne K. H. de Boer

**Affiliations:** 1 Department of Gastroenterology and Hepatology, Amsterdam UMC, Vrije Universiteit Amsterdam, 1081 HV Amsterdam, The Netherlands; 2 Amsterdam Gastroenterology Endocrinology Metabolism (AGEM) Research Institute, 1081 HV Amsterdam, The Netherlands; 3 Cancer Center Amsterdam, Research Program, 1081 HV Amsterdam, The Netherlands; 4 Department of Laboratory Medicine, Amsterdam University Medical Centre, 1105 AZ Amsterdam, The Netherlands; 5 Amsterdam UMC Location University of Amsterdam, Medical Library AMC, Meibergdreef 9, 1105 AZ Amsterdam, The Netherlands; 6 Department of Paediatric Gastroenterology, Emma Children’s Hospital, Amsterdam UMC, Vrije Universiteit Amsterdam, 1081 HV Amsterdam, The Netherlands; 7 Department of Paediatric Gastroenterology, Emma Children’s Hospital, Amsterdam UMC, Academic Medical Centre, 1105 AZ Amsterdam, The Netherlands; 8 Department of Gastroenterology and Hepatology, Amsterdam UMC, University of Amsterdam, 1081 HV Amsterdam, The Netherlands

**Keywords:** Non-invasive biomarker, Colorectal cancer, Sensitivity, Specificity, Amino acids

## Abstract

**Background:**

Metabolomics offers novel insights into metabolic alterations in colorectal cancer (CRC), including changes in amino acid profiles. Several studies have reported differences between CRC patients and controls, suggesting potential diagnostic utility.

**Purpose:**

To evaluate evidence on amino acid alterations in CRC and advanced precursors across biological matrices and their potential as non-invasive biomarkers.

**Method:**

A comprehensive search of MEDLINE, EMBASE, and Cochrane CENTRAL identifi ed 77 studies analysing amino acids in faeces, urine, serum, plasma, tissue, and saliva.

**Results:**

Few studies included advanced adenomas and none assessed advanced serrated polyps. Results were heterogeneous across matrices, except for tissue, where most amino acids were consistently upregulated in CRC. Reported diagnostic performance varied widely (AUC 0.28–0.91), with limited external validation.

**Conclusion:**

Overall, amino acids show limited standalone diagnostic value but may enhance multi-metabolitepanels. Standardisation, inclusion of early lesions, and robust validation are essential for future biomarker research.

**Supplementary Information:**

The online version contains supplementary material available at 10.1007/s12094-026-04278-9.

## Introduction

Metabolomics has emerged as a powerful tool in cancer research, enabling the comprehensive profiling of metabolites that reflect cellular processes in health and disease. One of the key insights from this field is that cancer cells, including those in colorectal cancer (CRC), undergo substantial metabolic reprogramming to support uncontrolled growth and proliferation, known as hallmarks of cancer [1]. These alterations include shifts in energy metabolism, biosynthetic demands and redox balance. In CRC, metabolic changes may also be modulated by the gut microbiota, which can influence the production and degradation of the metabolites within the intestinal lumen [2–4].

CRC arises from precursor lesions such as advanced adenomas and advanced serrated polyps through the adenoma-carcinoma (85%) or serrated pathway (15%), respectively [5]. CRC and those precursor lesions are collectively referred to as advanced colorectal neoplasia. Since the five-year survival rate drops from 90% for stage I to 14% for stage IV disease, early detection and removal of CRC and precursor lesions can lower incidence and improve survival [6, 7]. Faecal immunochemical testing (FIT) is widely used in European population-based screening programs to identify individuals at high-risk for CRC or its advanced precursors. However, FIT has limited sensitivity for advanced adenomas and serrated polyps, combined with modest specificity for CRC, leading to missed lesions and a high number of unnecessary colonoscopies [8, 9]. This highlights the need for novel, non-invasive biomarkers that can improve screening accuracy.

Among the many metabolic changes identified in CRC, alterations in amino acid concentrations and pathways have emerged as one of the most frequently reported findings. Differences in amino acid profiles between CRC patients and controls have been reported across matrices such as faeces, serum, and urine, indicating potential as non-invasive biomarkers [10–12]. However, a systematic, cross-matrix comparison of these amino acid alterations is currently lacking. This gap limits the ability to integrate findings across matrices and to assess the robustness and translational relevance of amino acids as reliable biomarkers for advanced colorectal neoplasia.

This systematic review aimed to summarise current evidence on amino acid profile alterations in patients with advanced colorectal neoplasia and controls, aiming to identify neoplasia-specific profiles. In addition, we evaluated the extent of overlap in differential amino acids between matrices and assessed the diagnostic potential of amino acids as non-invasive biomarkers for advanced colorectal neoplasia detection.

## Method

The protocol for this systematic review was prospectively registered in the PROSPERO database (CRD42022347826) and findings have been described in accordance with Preferred Reporting Items for Systematic reviews and Meta-Analyses (PRISMA) statement.

### Information sources and search strategy

Literature search strategies were developed by an information specialist and the first author (FJ and RO) using medical subject heading (MeSH) and text words related to ‘advanced adenoma’, ‘advanced serrated polyp’, ‘colorectal cancer’, ‘amino acids’, and ‘biomarker’. Articles were identified from MEDLINE, EMBASE and Cochrane Central Register of Controlled Trials on 29 November 2022. The full search is presented in Supplementary Table 1. The search was not limited to a specific language or date. Reference lists of included studies or relevant reviews identified through the search were scanned to check for any further eligible publications. An updated search was performed on 22-05-2025. To identify any significant new publications since the last update, a targeted screening of PubMed was conducted for the period from May to December 2025.

### Selection process and patient population

The selection process was performed by two independent reviewers (RO and AH). Studies were included if they met the following criteria: (i) studies consisting of patients of 18 years or above with advanced adenoma, advanced serrated polyp or CRC; (ii) the diagnosis of AA, advanced serrated polyp or CRC was based on endoscopy combined with histopathology; (iii) studies on amino acid analysis for diagnostic biomarker discovery. The following definitions were used to classify advanced colorectal neoplasia: advanced adenomas were defined as conventional adenomas measuring ≥ 10 mm in diameter, and/or exhibiting villous histology (≥ 25% villous component), and/or high-grade dysplasia; advanced serrated polyps included sessile serrated lesions or hyperplastic polyps ≥ 10 mm, sessile serrated lesions with dysplasia or traditional serrated adenomas; and CRC was defined as histologically confirmed adenocarcinoma of the colon or rectum, irrespective of stage at diagnosis. A list of included amino acids is presented in Supplementary Table 2. Studies were excluded if they: involved patients with hereditary risk for colorectal neoplasia or patients with polyposis syndromes (e.g., Lynch syndrome, familial adenomatous polyposis, or serrated polyposis syndrome (SPS)); were non-original research or case reports; or lacked information on how controls were classified (e.g., absence of information on diagnostic work-up). Although the initial search strategy was broad and included general biomarker terms, we restricted the review during full-text screening to diagnostic applications to maintain a feasible and coherent scope for the review.

### Data extraction and outcomes

From each study we extracted author, year, design, population, demographics, matrix, analytical method, amino acids measured, direction of change, effect size, *p* value, and histopathological data on advanced adenoma, advanced serrated polyps and CRC. Diagnostic performance (AUC, sensitivity, specificity) was extracted when reported for individual amino acids or panels consisting exclusively of predefined amino acids. If multiple time points or subgroups were reported, we prioritised baseline and predefined subgroup analyses. Data extraction was performed by RO using predefined forms and verified by AH to reduce errors. Missing or additional data were requested from study authors or retrieved from cited sources. Unresolved disagreements were resolved by a third author (SB). Results were synthesised using descriptive and tabular methods. Due to heterogeneity in study design, analytical methods, outcomes, and biospecimen types, no meta-analysis was performed. Findings were summarised narratively by matrix, lesion type, outcome type (e.g. concentration differences, diagnostic performance), and analytical approach (targeted or untargeted). To support interpretation, a forest plot was used to display AUCs, and a visual summary was generated to show the direction and consistency of amino acid changes across studies.

### Quality assessment

The methodological quality of the included studies was assessed using the Newcastle–Ottawa Scale (NOS). For case–control studies, the original NOS tool was used, evaluating studies across three domains: selection, comparability, and exposure, with a maximum score of nine points. For cross-sectional studies, the modified version of the NOS as described by Modesti et al. (2016) was applied [13]. This adaptation, specifically developed for non-cohort observational designs, evaluates methodological quality across the domains of selection, comparability, and outcome, with a maximum score of ten points. Both versions are provided in the supplementary material page 3 and 4. Study assessment was performed by RO. In cases requiring clarification, a second author was consulted.

### Statistics

If effect size as fold changes were not reported in the original publication, they were calculated manually based on the reported means or medians. For each study, it was clearly indicated whether fold changes were extracted or calculated in the corresponding table. For the study by Bosch et al., raw data were publicly available and used to perform additional statistical analyses [14]. Mann–Whitney U tests were conducted to compare CRC versus controls, advanced adenomas versus controls, and CRC plus advanced adenomas versus controls using RStudio (version 4.4.3).

## Results

### Overview of included studies

#### Study selection

**Fig. 1:**
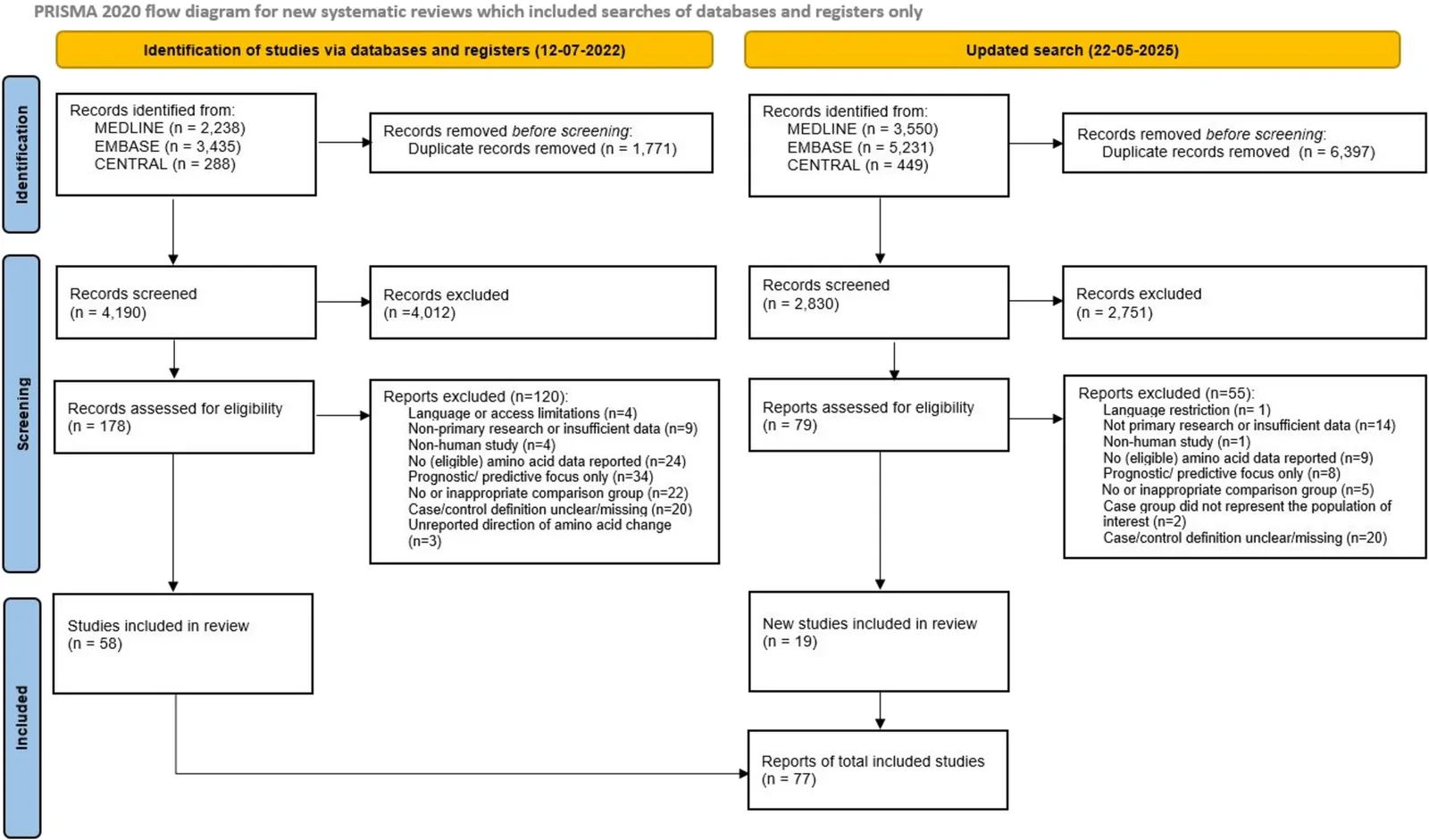
Preferred reporting items for systematic reviews and meta-analyses (PRISMA) flow diagram. *CENTRAL* Cochrane Central Register of Controlled Trials

The complete screening process is outlined in the PRISMA 2020 flow diagram (Fig. 1). The initial literature search yielded 5961 records. After deduplication, 4190 titles and abstracts were screened, leading to 178 full-text assessments. An updated search identified 2839 additional records, of which 79 were reviewed in full-text, resulting in a total 266 articles undergoing full-text evaluation. Most excluded studies were prognostic or predictive in nature (*n* = 42), lacking clear definitions of cases and controls (*n* = 40), or not meeting inclusion criteria for amino acids (*n* = 33). Additional exclusions were made for studies addressing alternative comparisons (*n* = 27), language/access issues (*n* = 5), non-primary research or insufficient data (*n* = 9), non-human studies (*n* = 5), and unclear outcomes due to unreported amino acid changes (*n* = 3). The targeted screening of PubMed did not identify any additional eligible studies that substantially affected the results or conclusions of the present review. In total, 77 articles were included in this systematic review.

#### Study characteristics

**Fig. 2:**
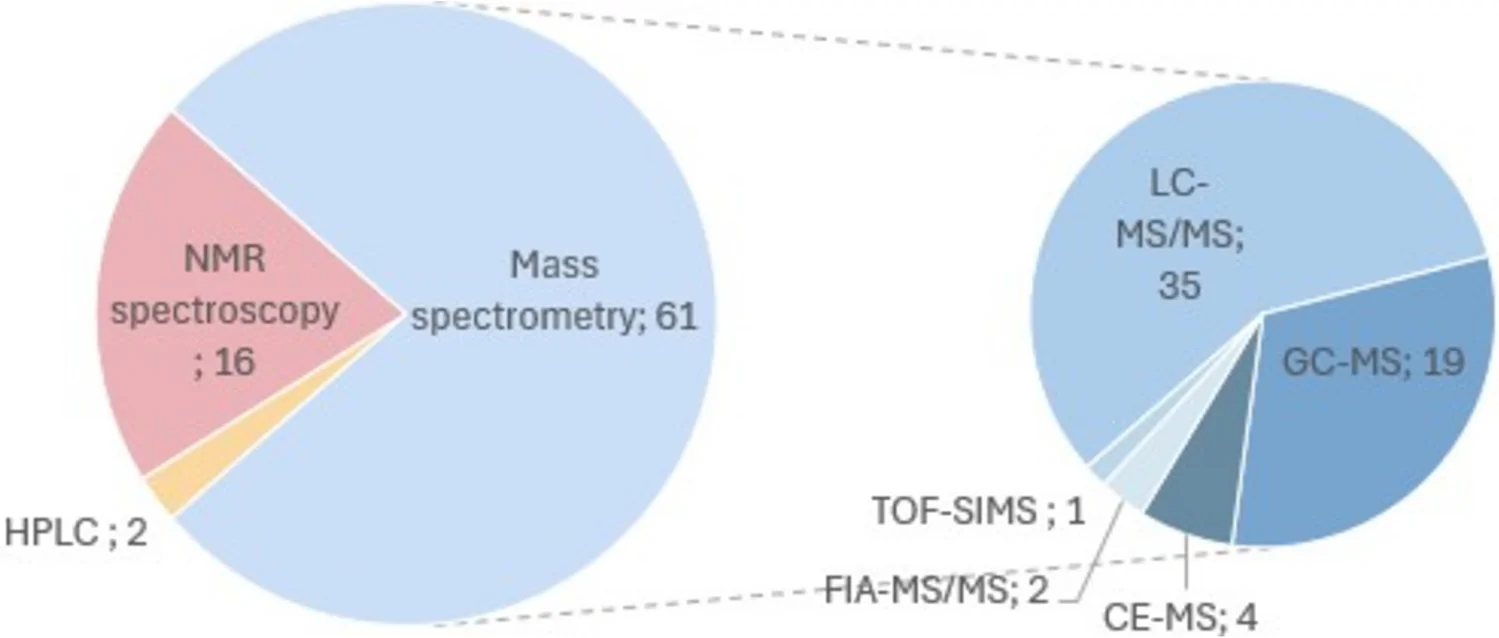
Distribution of analytical techniques used for amino acid measurement. Pie chart illustrating the distribution of analytical methods applied across the included studies. A detailed breakdown of mass spectrometry-based techniques is provided in the adjacent inset. The number following each technique indicates the number of studies that employed the respective method. The total number of methods exceeds the number of included studies, as two studies employed two distinct analytical approaches. *CE-MS* Capillary Electrophoresis–Mass Spectrometry; *FIA-MS/MS* Flow Injection; Analysis–Tandem Mass Spectrometry; *GC–MS* Gas Chromatography–Mass Spectrometry; *HPLC* High-Performance Liquid Chromatography; *LC–MS/MS* Liquid Chromatography–Tandem Mass Spectrometry; *NMR* spectroscopy Nuclear Magnetic Resonance Spectroscopy; *TOF–SIMS* Time-of-Flight Secondary Ion Mass Spectrometry

Details on study characteristics are summarized in Table 1. A total of 77 studies published between 1996 and 2025 were included in this review. The majority were case–control studies, alongside a smaller number of cross-sectional cohort designs. Most studies were conducted in China (*n* = 35), followed by European countries (*n* = 15), Japan (*n* = 8), the United States (*n* = 7), and South Korea (*n* = 3). Fewer studies were conducted in Singapore (*n* = 3), Canada (*n* = 3), and Iran, Egypt, Nigeria, and Turkey (each *n* = 1). All studies involved adults with colorectal cancer (77 studies; total CRC cases = 6263), with the number of cases per study ranging from 8 to 437. Six studies included individuals with advanced adenomas (*n* = 351), with sample sizes ranging from 10 to 159. A total of 56 studies included a healthy control group (*n* = 6283), with the number of controls per study ranging from 3 to 995. No studies were identified that included adults with advanced serrated polyps. Various biological matrices were described across the included studies, including faeces (*n* = 18), urine (*n* = 8), serum (*n* = 20), plasma (*n* = 8), tissue (*n* = 23), saliva (*n* = 1), serum-derived exosomes (*n* = 1), and microbiome-derived vesicles (*n* = 1). Three main categories of analytical techniques were employed across the included studies: mass spectrometry-based approaches (*n* = 61), nuclear magnetic resonance (NMR) spectroscopy (*n* = 16), and high-performance liquid chromatography (HPLC; *n* = 2). Among the mass spectrometry-based methods, liquid chromatography–mass spectrometry (LC–MS/MS; *n* = 35) and gas chromatography–mass spectrometry (GC–MS; *n* = 19) were most frequently used, followed by capillary electrophoresis–mass spectrometry (CE-MS; *n* = 4), flow injection analysis–tandem mass spectrometry (FIA-MS/MS; *n* = 2), and time-of-flight secondary ion mass spectrometry (TOF–SIMS; *n* = 1) (Fig. 2). Supplementary Table 3 provides an overview of the distribution of primary analytical methods across different matrices and their frequency of use in targeted versus untargeted studies. Although all included methods were classified under these overarching categories, a range of analytical variants was applied, differing in aspects such as instrumentation and the use of commercial kits. Details of specific analytical configurations and instrumentation are provided in Supplementary Tables 4–6.

**Table 1:** Study characteristics^a^

Author [year] refs	Study design, country	Study group	number	Gender, m/f	Age (y)	Diagnosis (case|control)	CRC TNM stage (*n*)	Matrix
							0|I|II|III|IV	
Bosch, S. [2022] [14]	Case–control study, the Netherlands	CRC|AA|Ctrl	12|10|20	6/6|9/1|14/6	67 [60–71]|71 [70–73]|67 [62–75] ^b^	C|C	no data	Faeces
Coker, O. [2022] [10]	Cross-sectional cohort study, China	CRC|Ctrl	118|128	64/54|59/69	73.21 ± 10.37|64.03 ± 6.84 ^c^	C|C	no data	Faeces
Du, X. [2022] [28]	Case–control, China	CRC|Ctrl	30|33	12/18|13/20	56.67|59.27 ^c^	C|C	0|2|12|14|2	Faeces
Erben, V. [2021] [22]	Cross-sectional cohort study, Germany	CRC + AA|Ctrl	12 + 159|229	103/68|123/106	61.1 ± 8.6|60.9 ± 8.0 ^c^	C|C	no data	Faeces, urine and plasma
Kim, M. [2020] ^20^	Case–control, USA	AA|Ctrl	102|102	62/40|62|40	50-59y 17, 60-69y 50, > 70y 35 | 50-59y 18, 60-69y 49, > 70y 35	C|C	no data	Faeces
Kong, C. [2022] [41]	Case–control, China	LO-CRC|Ctrl	130|97	81/49|42/55	62.32 ± 7.96 | 61.56 ± 7.35 ^c^	C|C	0 + I 18|46|52|14	Faeces
		EO-CRC|Ctrl	114|100	57/57|47/53	39.96 ± 7.01 |39.00 ± 7.31 ^c^		0 + I 15|28|49|22	
Kulecka, M. [2024] [53]	Case–control, Poland	CRC|Ctrl	40|40	20/20|20/20	♀: 66[36–82], ♂:67[35–82] | ♀: 68[49–79], ♂:61[50–81]	C|Screening	no data	Faeces
Le Gall, G. [2018] [54]	Case–control, UK	Set 1: CRC|Ctrl	20|20	12/8|12/8	67[61–72]|67[60–74] c	C|C	Duke 3 A, 7B, 5C	Faeces
		Set 2: CRC|Ctrl	30|29	21/9|21/9	66[60–74]|66[60–74] c		Duke 3 A, 2B, 11C	
Liang, L. [2024] [40]	Case–control, China	RCC|LCC|Ctrl	63|79|88	no data	no data	C|C	no data	Faeces
Lin, Y. [2019] [55]	Case–control, China	CRC|Ctrl	50|50	sex matched, no data	age matched, no data	C|ME	no data	Faeces
		CRC|DNT|ANT	70	no data	no data	H	No data	Tissue
Lin, Y. [2016] [24]	Case–control, China	CRC|Ctrl	68|32	36/32|15/17	56 ± 21|57 ± 23	H|ME	0|I + II 20|25|23	Faeces
Monleon, D.[2009] [56]	Case–control, Spain	CRC|Ctrl	21|11	7/14|2/9	65[32–79]|59[31–59)] ^b^	C|C	no data	Faeces
Ramzy, A. [2025] [57]	Case–control, Egypt	CRC|Ctrl	20|20	20/0|20/0	53.9 ± 4.8|53.5 ± 3.7	C|BT/C/I	0|2|1|4|3	Faeces
Sun, X. [2020] [58]	Case–control, China	CRC|Ctrl	46|38	32/14|24/14	63.63 ± 11.39|6.85 ± 10.99 ^c^	C|C	no data	Faeces
Telleria, O. [2022] [19]	Case–control, Spain	CRC|AA|Ctrl	40|40|40	20/20|20/20|20/20	69[38–91]|71[53–86]|76[40–88] ^b^	C|C	no data	Faeces
Yachida, S. [2019] [2]	Cross-Sectional Cohort Study, Japan	CRC|AA|Ctrl	148|30|149	88/60|15/15|86/63	I + II 63.58 ± 8.84, III + IV 59.5 ± 11.46|63.73 ± 8.77 | 64.11 ± 10.9	C|C	30|51|29|44|24	Faeces
Yang, Y. [2019] [59]	Case–control, China	CRC|Ctrl	50|50	26/24|17/33	< 60y: 14, > 60: 36|< 60: 20, > 60: 30	C|C	2|9|13|16|10	Faeces
Zhang, Q. [2024] [60]	Cross-Sectional Cohort Study, China	CRC|Ctrl	245|244	153/92|107/137	54.10 ± 13.23|49.94 ± 13.56 ^c^	C|C	0|I + II 123|III-IV 121	Faeces
Kim, D. [2020] [15]	Case–control, Korea	CRC|Ctrl	32|40	20/12|22/18	64[45–80]|64.5[49–78] ^c^	C|ME	1|7|12|9|3	MEV
Yagin, F. ^d^ [2023] [61]								
Deng, L. [2019] [62]	Case–control study, Canada	CRC-CAD|CRC-MSKCC|Ctrl	121|50|171	68/59|24/26|100/71	67.4 ± 10.9|63.8 ± 12.5|58.9 ± 5.6 ^c^	C|C|C	CAD: 3|16|30|51|21	Urine
							MSKCC: 0|14|20|6|10	
Deng, Y. [2020] [16]	Case–control study, China	CRC|Ctrl	139|50	91/48|28/22	63[36–87]|61[47–89] ^b^	H|ME	8|26|42|50|13	Urine
Deng, W. [2024] [11]	Case–control study, China	CRC|Ctrl	155|115	63/52|145/10	64|46 ^b^	H|ME	0|14|31|48|22	Urine
Kim, E. [2019] [23]	Case–control study, Korea	CRC|AA|Ctrl	24|36|156	no data|76/80	no data|52 [22–76] ^b^	C|C	24|0|0|0|0	Urine
Ning, W. [2021] [17]	Case–control study, China	CRC|Ctrl	163|111	103/60|69/42	66 < 60, 97 > 60,|61 < 60, 50 > 60	C|ME	0 | 0 | 65 | 74 | 24	Urine
Wang, Z. [2017] [18]	Case–control study, China	CRC|Ctrl	23|40	11/12|19/21	61[27–84]|59[28–78] ^b^	C|NHC + no GI symptoms	only stage I and II	Urine
Zhang, L. [2023] [63]	Cross-sectional cohort study, Nigeria	CRC|Ctrl	169|194	95/74|125/69	55[19–73]|56[21–74] ^b^	C|C	No data	Urine
Bednarz-Misa, I. [2020] [32]	Case–control, Poland	CRC|Ctrl	137|54	72/65|24/30	63.6 ± 10.9|60.5 ± 13 ^c^	C|ME/BT	5|17|52|46|17	Serum
Deng, L. [2016] [64]	Cross-sectional cohort study, USA	CRC|Ctrl	28|55	16/12|25/30	55.3[27–86]|52.8[21–74] ^c^	C|C	0|I + II 3|8|17	Serum
Farshidfar, F. [2016] [65]	Case–control, Canada	CRC|Ctrl	320|254	201/119|148/106	I 68.6 ± 10.6, II 68.6 ± 12.4, III 64.9 ± 13.1, IV 63.1 ± 11.5| 61.7 ± 9.3 ^c^	C|C	0|47|60|71|142	Serum
Farshidfar, F. [2018] [66]	Case–control, Canada	CRC|Ctrl	62|81	46/16|58/23	I 76.9 ± 6.1, II 67.3 ± 9.8, III 62.8 ± 14.8, IV 60.5 ± 9.9 | 60.5 ± 6.7 ^c^	C|C	0|8|8|8|38	Serum
Gu, J. [2019] [67]	Case–control, China	CRC|Ctrl	40|38	27/13|21/17	60[25–82]|55[35–77] ^b^	C|C	0|7|9|14|14	Serum
Guo, J. [2023] [68]	Case–control, China	CRC|Ctrl	8|14	4/4|7/7	46.9 ± 7.7|43.4 ± 6.1 ^c^	C|C	no data	Serum
Huang, H. [2002] [69]	Case–control, UK	CRC|Ctrl	66|37	39/27|13/27	66[61–73]|69[64–74] ^e^	C|NHC	no data	Serum
Ikeda, A. [2011] [70]	Case–control, Japan	CRC|Ctrl	12|12	8/4|5/7	71.3[63–83]|58.5[45–74] ^b^	C|BT/C/I	0|3|4|5|0	Serum
Leichtle, A. [2012] [29]	Case–control, Germany	CRC|Ctrl	59|58	37/22|26/32	59 [45–90]|58 [38–75] ^b^	C|BT/ME	0|5|20|16	Serum
Li, J. [2019] [12]	Case–control, China	Screening: CRC|Ctrl	120|120	73/47|73/47	63.1 ± 13.1|61.7 ± 12.2 ^c^	H|ME	0|11|35|62|12	Serum
		Validation: CRC|Ctrl	437|580	252/185|298/282	59.1 ± 12.5|58.1 ± 11.4		0|76|126|153|82	
Long, Y. [2017] [71]	Case–control, China	CRC | Ctrl	30|30	18/12|18/12	53.97 ± 13.46|55.23 ± 10.46 ^c^	H|NHC	0|I + II 20|25|23	Serum
Nishiumi, S. [2012] [27]	Case–control, Japan	Training: CRC|Ctrl	60|60	39/21|39/21	67.7[36–88]|64.5[39–88] ^c^	H|BT/C/I	12|12|12|12|12	Serum
		Validation: CRC|Ctrl	59|63	30/29|32/31	64.8[31–84]|62.8[47–73] ^c^		15|11|3|11|19	Serum
Qiu, Y. [2009] [72]	Case–control, China	CRC|Ctrl	64|65	35/29|34/31	59[42–74]|55[42–69] ^b^	H|ME	0|9|27|20|8	Serum
Tan, B. [2013] [73]	Case–control, China	CRC|Ctrl	62|62	34/28|28/34	60.1 [24 − 82]|59.4 [31 − 75] ^c^	C|ME	0|16|25|17|4	Serum
Tevini, J. [2022] [21]	Case–control, Austria	Training: CRC|AA|Ctrl	18|28|36	11/7|14/14|18/18	67 ± 12|60 ± 10|53 ± 8	C|C	no data	Serum
		Validation: CRC|Ctrl	48|29	31/17|3/26	69 ± 10|68 ± 7			
		Validation: AA|Ctrl	48|28	26/22|28/0	66 ± 10|66 ± 5			
Tristán, A. [2023] [26]	Case–control, Spain	CRC|Ctrl	57|26	29/28|14/12	60.0 ± 5.33|65.5 ± 6.83 ^c^	C|ME	0|0|0|0|57	Serum
Uchiyama, K. [2017] [39]	Case–control, Japan	CRC|Ctrl	56|60	25/28|30/30	I 70.4 ± 8.2, II 69.3 ± 9.6, IIIa 71 ± 6.7, IIIb 71.3 ± 10.4, IV 70.7 ± 10|67.7 ± 9.2 ^c^	C|C	0|14|14|14|14	Serum
Wu, J. [2020] [74]	Case–control, China	CC|RC|Ctrl	22|23|45	15/7|16/7|31/14	66.49[49–84]|64.41[49–84]|68.48[54–80]^c^	C|BT	no data	Serum
Zhu, J. [2014] [75]	Cross-sectional cohort study, USA	CRC|Ctrl	66|92	30/36|45/47	58[27–88]|57[18–80] ^b^	C|C	0|I + II 21|17|28	Serum
Eylem, C. [2020] [76]	Case–control, Turkey	CRC|Ctrl	8|8	6/2|no data	56.5[53–71]|no data ^b^	H|ME	0|2|2|1|3	Serum exosomes
Coradduzza, D. [2022] [77]	Case–control, Italy	CRC|Ctrl	50|52	33/17|no data	7.74 ± 10.44|55.42 ± 5.14 ^c^	H|ME	1|10|13|17|7|2	Plasma
Geijsen, A. [2019] [78]	Case–control, Germany + Austria	Discovery: CRC|Ctrl	180|153	114/66|59/94	66.0[58.0–73.0]|51.0[42.0–63.0] ^e^	H|NHC	7|34|66|47|25	Plasma
		Replication: CRC|Ctrl	88|200	60/28|130/70	70.0[60.0–76.0]|64.0[57.0–74.0] ^e^		0|30|17|18|12	
Miyagi, Y. [2011] [30]	Case–control, Japan	CRC|Ctrl	199|995	126/73|570/425	CRC 63.7 ± 9.5|62.4 ± 9.5 ^c^	C|ME	8 | 63 | 48 | 59 | 19	Plasma
Nishiumi, S. [2017] [31]	Case–control, Japan	CRC|Ctrl	282|291	170/112|178/113	67 ± 9.02|66.8 ± 7.94 ^c^	C|CRC screening	79|80|123|0|0	Plasma
Okamoto, N.[2009] [79]	Case–control, Japan	CRC|Ctrl	103|62	42/20|52/51	62.7 ± 9.5|57.6 ± 6.1 ^c^	H|ME	2|9|22|22|6	Plasma
Rodriguez-Tomas, E. [2021] [80]	Case–control, Spain	RC|Ctrl	32|48	19/29|22/10	67.7 ± 9.5|41.9 ± 10.0 ^c^	H|ME	0|0|5|27|0	Plasma
Sun, Y. [2024] [81]	Case–control, China	CRC|Ctrl	111|119	64/47|52/67	61.90 ± 11.49|43.76 ± 11.60	C|C	3|21|17|36|18	Plasma
Arima, K. [2020] [82]	Case–control, paired, USA	CRC + paired normal	11	no data	no data	H	no data	Tissue
Cai, Y. [2020] [83]	Case–control, USA	CRC|normal ^f^	39|39	20/19|27/12	69.5 ± 8.5|67.4 ± 12.4 ^c^	H	0|13|14|12|0	Tissue
Cai, R. [2025] [84]	Case–control, paired, China	CRC + paired normal	106	51/55	< 60: 44|≥ 60: 62	H	0|6|19|43|32	Tissue
Chan, E. [2009] [85]	Case–control, paired, Singapore	CRC + paired normal	31	18/13	67 ± 13 ^c^	H	0|3|10|13|5	Tissue
Cho, K. [2021] [86]	Case–control, paired, Korea	CRC + paired normal	90	45/45	no data	H	no data	Tissue
Denkert, C. [2008] [87]	Case–control, Germany	CRC|Ctrl ^g^	27|18	12/15	no data	H	0|3|9|14|1	Tissue
Feizi, H. [2025] [88]	Case–control, Iran	CRC|Ctrl	14|20	7/7|10/10	40-49y 2, 50-59y 1, 60-69y 4, 70-79y 7 | 40-49y 2, 50-59y 4, 60-69y 6, 70-79y 8	H|H	no data	Tissue
Gao, P. [2016] [89]	Case–control, China	CRC + paired normal	11	no data	no data	H	no data	Tissue
Jiménez, B. [2013] [90]	Case–control, UK	CRC + paired normal	83|87 h	10/16	72[26–87] ^b^	H	0|2|10|14|0	Tissue
Kinross, J. [2017] [91]	Case–control, paired, UK	CRC + paired normal	18	10/8	76[55–85] ^b^	H	No data	Tissue
Long, Z. [2020] [92]	Case–control, paired, China	CRC + paired normal	51	24/26	57[22–79] ^b^	H	0|4|21|15|4	Tissue
Lv, W. [2020] [93]	Case–control, paired, China	CRC|DNT|ANT	22	13/9	♂ 64.46 ± 3.49, ♀ 60.44 ± 4.00 ^c^	H	No data	Tissue
Mal, M. [2012] [94]	Case–control, paired, Singapore	CRC + paired normal	31	18/13	67 ± 13 ^c^	H	0|4|10|13|5	Tissue
Manna, S. [2014] [95]	Case–control, paired, USA	CRC + paired normal	39	19/20	69 ± 15.3 ^b^	H	0|I + II 21|III + IV 18	Tissue
Moreno, A. [1996] [96]	Case–control, partly paired, Spain	CRC + paired normal	16|10	11/5	65 ± 11 ^c^	H	0|5|4|6|1	Tissue
Ning, W. [2017] [25]	Case–control, paired, China	CRC + paired normal	20	13/7	8 < 60 years 12 > 60 years	H	0|0|8|9|3	Tissue
Phua, L.C. [2014] [97]	Case–control, paired, Singapore	CRC + paired normal	11	7/4	64.5[56–80] ^c^	H	0|0|6|5|0	Tissue
Qiu, Y. [2014] [98]	Case–control, USA + China	[Set 1] CRC + partly paired normal	85|55	49/36	57[31–79] ^b^	H	0|7|35|37|6	Tissue
		[Set 2] CRC + paired normal	23	10/13	61[40–75] ^b^		0|3|9|10|1	
		[Set 3] CRC + paired normal	65	43/22	61[34–84] ^b^		0|11|22|21|11	
		[Set 4] CRC + paired normal	29|27	8/12	59[35–81] ^b^		1|2|2|14|1	
Rao, J. [2024] [99]	Case–control, paired, China	CRC + paired normal	10	8/2	65[34–85] ^b^	H	0|1|2|6|1	Tissue
Wang, H. [2013] [100]	Case–control, partly paired, China	RC|Ctrl ^i^	127|43	69/58|16/27	55[28–86]|56[35–85] ^e^	H	0|35|37|37|18	Tissue
Wang, Q. [2020] [101]	Case–control, paired, China	CRC + paired normal	17	No data	No data	H	No data	Tissue
Zha, H. [2018] [102]	Case–control, paired, China	CRC + paired normal	18	9/9	61.35 ± 9.52 2	H	No data	Tissue
Su, H. [2025] [103]	Case–control, China	CRC|Ctrl	35|36	15/20|15/21	51.51 ± 11.47|41.39 ± 7.41 ^c^	C|C	0|7|12|10|6	Saliva

^a^*CRC* colorectal cancer, *AA* advanced adenoma, Ctrl control, *LO-CRC* late onset colorectal cancer (≥ 50 years), *EO-CRC* early onset colorectal cancer (< 50 years), *CC* colon cancer, *CRC-CAD* Colorectal cancer patients from the Edmonton region in Canada, *CRC-MSKCC* Colorectal cancer patients from the Memorial Sloan Kettering Cancer Centre, *RC* rectal cancer, *DNT* Distant normal issue, *ANT* adjacent noncancer tissues, *H* histopathology, *C* colonoscopy, *ME* medical examination, *MEV* microbial derived vesicles, *BT* Blood tests, *I* imaging, *NHC* no history of cancer, *LCC* Left sided colon cancer, *RCC* Right sided colon cancer

^b^ Median ± SD or [range]. ^c^ Mean(± SD) or [range]. ^d^ Yagin et al. performed a secondary analysis of publicly available data from Kim et al. to explore its diagnostic utility. ^e^ Median[IQR]. ^f^ Normal tissue was obtained from sites distant to the tumour at the resection margin, but not from the same CRC patients included in the primary comparison group. ^g^ Cases with paired normal and carcinoma tissue *n* = 15. ^h^ Derived from 26 patients. ^i^ Cases with paired normal and carcinoma tissue *n* = 43

#### Quality assessment

The NOS was applied to 70 case–control studies and seven cross-sectional studies (Supplementary Tables 7 and 8). In most studies, cases were clearly defined and diagnosed using colonoscopy. However, in 13 studies (17%), while CRC or polyps were confirmed endoscopically, the histological classification was incomplete, with unclear distinction between advanced and non-advanced adenomas, or between adenomas and serrated polyps. Therefore, only the CRC versus control comparison in these studies was included in the present review. In 27 case–control studies (39%), controls were not colonoscopy-confirmed but identified based on alternative criteria, including absence of symptoms, normal clinical assessment, or lack of cancer history (Table 1). Although this introduces a potential risk of undetected lesions, these individuals were considered sufficiently low-risk to be included in CRC versus control comparisons. Fifty-four studies (70%) adjusted for age, and 51 studies (66%) for additional confounders including, sex, BMI, ethnicity, or smoking status. Metabolomic and amino acid analyses were consistently performed between cases and controls. Exclusion criteria were clearly reported in all studies, however, no study met the NOS non-response criterium. Overall, methodological quality ranged from 6 to 8 of 9 stars.

### Differential amino acid profiles

#### Amino acid profiles in colorectal cancer versus controls

**Fig. 3:**
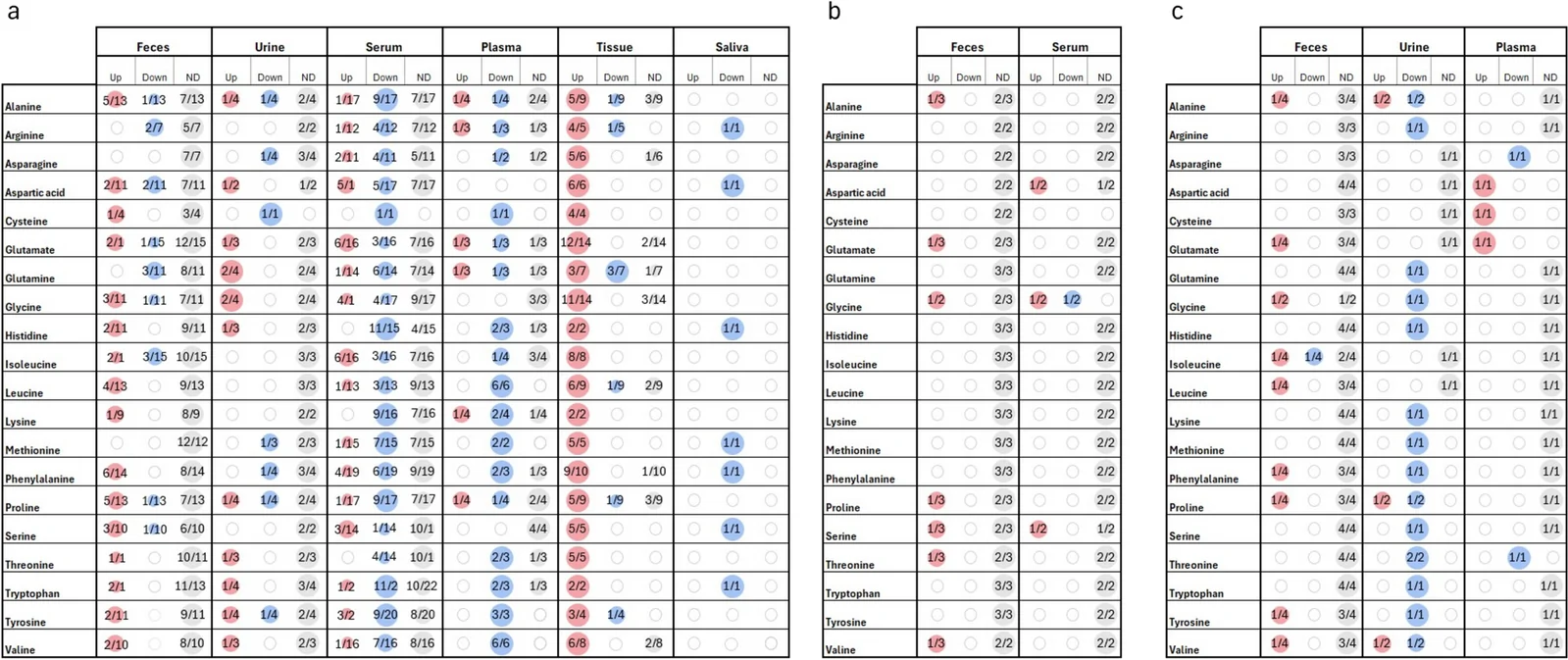
Directional changes in amino acid concentrations across matrices and comparisons, based on both targeted and untargeted approaches. Panels show results for **a** CRC versus controls, **b** advanced adenoma patients versus controls, and **c** advanced neoplasia patients versus controls. For each comparison, a matrix-specific summary is shown for the 20 amino acids included in this review. The Y-axis lists the amino acids; the X-axis indicates the biological matrices. Each circle reflects the number of studies reporting a significant increase (red), decrease (blue), or no significant difference (ND, grey) in amino acid levels. Circle size corresponds to the number of studies, with larger circles indicating greater consistency. Numbers within the circles denote how many studies reported the respective outcome out of the total number of studies assessing that amino acid in that matrix. Deng et al. (2024) reported L- and D-enantiomers separately; only amino acids showing significant changes in both forms were included for consistency

Supplementary Table 4 summarises the full study findings for the CRC versus controls comparison. A total of 17 studies investigated faecal amino acid profiles, encompassing 21 cohorts (CRC patients: *n* = 1335; controls: *n* = 1170). The majority of studies (12 out of 17; 71%) employed mass spectrometry, including LC–MS (*n* = 7), GC–MS (*n* = 4), and CE–MS (*n* = 1). Additionally, four studies (24%) used NMR spectroscopy, and one study applied HPLC. For nearly all amino acids, most studies found no significant differences in faecal concentrations between CRC patients and controls. Nonetheless, with the exception of four amino acids, at least one study per metabolite report a significant increase in CRC, particularly for alanine, phenylalanine, and proline (≥ 5 studies) (Fig. 3a, Supplementary Table 4). Nine amino acids were reported as significantly decreased in CRC patients in some studies, however, non-significant findings were more common across the literature. Due to these inconsistencies, no clear CRC-specific faecal amino acid pattern could be identified. This lack of consistency persisted when we analysed targeted and untargeted studies separately, with most studies again reporting non-significant differences (Supplementary Fig. 1a). One untargeted study specifically analysed microbial extracellular vesicles (MEVs) isolated from faeces and identified elevated levels of leucine, isoleucine, alanine, and lysine in CRC patients, which partly align with results from faecal studies [15].

Serum amino acid profiles showed a similar degree of inconsistency as observed in faecal samples. However, amino acids in serum were more frequently found to be in CRC patients. In total, 20 studies investigated serum samples, encompassing 28 cohorts (CRC patients: *n* = 1764; controls: *n* = 1750). Mass spectrometry was the most commonly used analytical technique, applied in 18 of the 20 studies (90%), including LC–MS (*n* = 8), GC–MS (*n* = 7), FIA-MS (*n* = 2) and CE–MS (*n* = 1). NMR spectroscopy was used in three studies (15%), while one study employed HPLC. For each of the 20 amino acids, at least one study reported reduced concentrations in CRC. Alanine, histidine, lysine, proline, tryptophan, and tyrosine were more often found to be downregulated than non-differential, although the difference was marginal. For 16 amino acids, increased levels were also described; glutamate and isoleucine were elevated in six studies, whereas seven other studies found no significant difference for these same metabolites. When analysing targeted and untargeted studies separately overall patterns remained largely consistent (Supplementary Fig. 1). However, targeted studies more frequently identified elevated levels of aspartic acid, glutamate, and isoleucine. In contrast, amino acids that were more commonly downregulated overall, including alanine, lysine, proline, tryptophan, and tyrosine, were more often classified as non-differential in the targeted analyses.

Seven studies investigated plasma amino acid profiles (eight cohorts, CRC patients: *n* = 1045; controls: *n* = 1920), all employing mass spectrometry (LC–MS *n* = 5, GC–MS *n* = 2). Overall, most amino acids were reported as either decreased or non-differential between CRC patients and controls (Fig. 3a). Notably, cysteine, leucine, methionine, tyrosine, and valine were consistently found to be downregulated in CRC patients across all studies. Stratification by analytical approach (targeted vs. untargeted) did not reveal substantial differences in overall findings (Supplementary Fig. 1).

Urine samples were analysed in six studies (six cohorts, CRC patients: *n* = 696; controls: *n* = 687), with mass spectrometry being the predominant technique (5/6 studies; 83%), including LC–MS (*n* = 4) and CE–MS (*n* = 1). NMR spectroscopy was applied in one study (17%). Most amino acids were reported as either non-differential or differentially altered in only one or two studies (Fig. 3a). Compared to other matrices, urine-based studies revealed more distinct patterns by analytical approach: targeted studies mainly reported no significant differences, while untargeted studies identified various altered amino acids (Supplementary Fig. 1). However, none of the three untargeted studies identified the same amino acids as differential [16–18]. This finding should be interpreted with caution, as selective reporting of only significant metabolites in untargeted studies may have influenced comparability.

Tissue-based analyses, comprising 23 studies (29 cohorts, CRC patients: *n* = 1101; controls: *n* = 1076), revealed the most consistent profile across all matrices, with all amino acids identified as upregulated in CRC tissue relative to normal mucosa (Fig. 3a). Only a small number of studies reported downregulated or non-differential levels. Glutamine was the exception, showing an equal number of studies reporting up- and downregulation, and one study reporting no significant difference. Several amino acids, including aspartic acid, cysteine, histidine, isoleucine, lysine, methionine, serine, threonine, and tryptophan, were consistently found to be significantly increased in all studies that assessed them. Similar to the other matrices, mass spectrometry was the predominant analytical technique, used in 16 of 23 studies (70%), including LC–MS (*n* = 7), GC–MS (*n* = 7), TOF–SIMS (*n* = 1), and CE–MS (*n* = 1). NMR spectroscopy was employed in 8 studies (35%).

Only one study investigated saliva samples using untargeted LC–MS and found decreased levels of arginine, aspartic acid, histidine, methionine, phenylalanine, serine, and tryptophan in 35 CRC patients compared to 36 controls. While some overlap with results from plasma and untargeted serum analyses, the overall pattern remained inconsistent and non-specific. Cysteine showed a relatively distinct trend, being consistently downregulated in urine, serum, and plasma, elevated in tissue, and largely non-differential in faecal samples. However, this observation is based on a limited number of studies per matrix and should be interpreted with caution.

#### Amino acid profiles in advanced adenoma versus controls

Supplementary Table 5 shows an overview of all findings in the advanced adenoma versus controls comparison. Four studies, comprising five independent cohorts, examined amino acid profiles in faecal (three studies, advanced adenoma patients: *n* = 154, controls: *n* = 162) and serum (one study comprising two cohorts, advanced adenoma patients: *n* = 76, controls: *n* = 64) samples from individuals with advanced adenoma compared to controls. Overall, most studies did not report significant differences in amino acid profiles, although a few individual amino acids were found to differ significantly (Fig. 3b, Supplementary Table 5). In one faecal cohort, targeted HPLC analysis showed elevated levels of alanine, glutamate, glycine, proline, serine, threonine, and valine in advanced adenoma patients compared to controls [14]. However, these findings were not in line with two untargeted studies, using UPLC-MS, which found no significant differences in the same amino acids [19, 20]. Similarly, in a targeted serum-based study using LC–MS in two independent cohorts, results for aspartic acid and serine were inconsistent: one cohort showed elevated levels in advanced adenoma patients, while the other reported no significant differences [21]. For glycine, opposing trends were observed, with upregulation in one cohort and downregulation in the other.

#### Amino acid profiles in advanced neoplasia versus controls

Seven studies assessed amino acid profiles in biological samples from individuals with CRC and advanced adenomas, collectively referred to as patients with advanced neoplasia, compared to controls. These included faecal samples (five studies, advanced neoplasia patients: *n* = 303; controls: *n* = 438), urine (two studies, advanced neoplasia patients: *n* = 219; controls: *n* = 385), and plasma (one study, advanced neoplasia patients: *n* = 159; controls: *n* = 229). All outcomes for the advanced neoplasia versus controls comparison are summarised in Supplementary Table 6. Overall, amino acid levels in faecal and plasma samples were predominantly non-differential between groups (Fig. 3c). In contrast, most urinary amino acids were decreased in advanced neoplasia patients, based on a targeted LC–MS approach [22]. However, inconsistent results were found for alanine, proline, and valine when compared to findings from an untargeted NMR-based study [22, 23]. Notably, threonine was found to be downregulated in both urine and plasma within the same study applying targeted LC–MS/MS [22]. Although certain amino acids were consistently reported as non-differential across sample types, the findings did not point to a clear amino acid signature for advanced neoplasia.

### Diagnostic potential

**Fig. 4:**
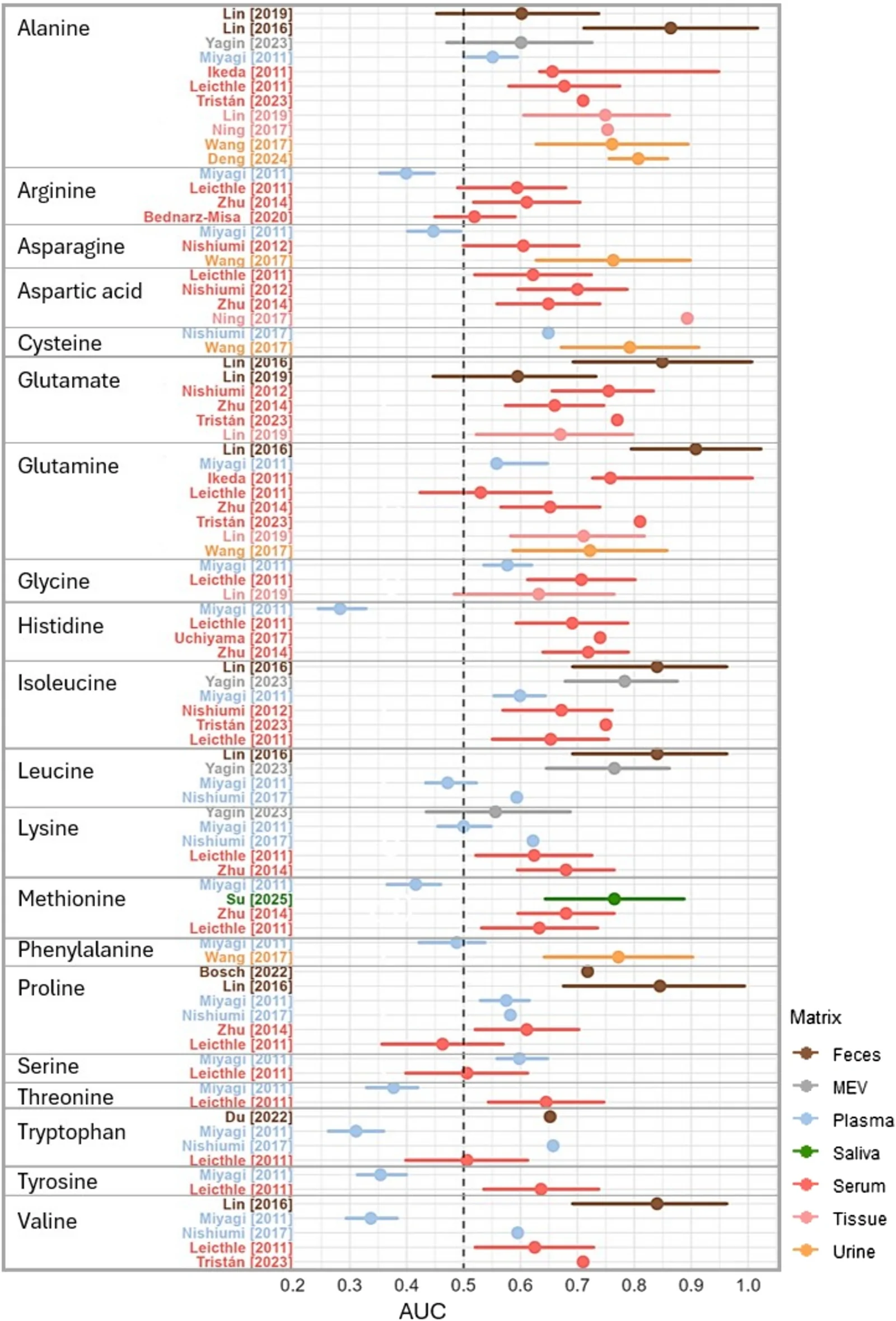
Overview of AUC values for amino acids by sample matrix for CRC detection. Forest plot displaying the reported AUC values for each amino acid in the comparison between CRC and controls. Each row represents a unique amino acid and biological matrix, showing the estimated AUC and its corresponding confidence interval, as reported in the referenced study (author and year in coloured y-axis labels). For some studies, no confidence interval was provided and only the AUC is shown. The colour of the data points reflects the matrix type and corresponds to the legend. *MEV* microbial extracellular vesicles

Several studies evaluated the diagnostic potential of individual amino acids for detecting CRC in various biological matrices: faeces (*n* = 3), urine (*n* = 1), serum (*n* = 6), plasma (*n* = 2), and tissue (*n* = 2). The diagnostic performance of individual amino acids across matrices is detailed in Supplementary Table 9, with most studies reporting area under the curve (AUC) values derived from ROC curve analysis. An overview of these AUCs is illustrated in Fig. 4. AUC values varied widely, ranging from poor to good discriminative ability depending on the matrix and amino acid. The highest values were observed in the faecal analysis by Lin et al., where alanine, glutamate, glutamine, isoleucine, leucine, proline, and valine achieved AUCs between 0.84 and 0.91 for the detection of stage I and II CRC [24]. Elevated AUCs were also described for tissue-based aspartic acid (AUC 0.89), serum glutamine (AUC 0.81), and urinary alanine (AUC 0.81) [11, 25, 26]. While most findings lacked validation, tissue-based aspartic acid was confirmed in an independent test set, though this was based on a limited sample size [25]. Additionally, Kim et al. reported a validated AUC of 0.783 (95% CI 0.714–0.839) for urinary alanine in detecting CRC and advanced adenomas [23]. No single amino acid consistently demonstrated good diagnostic performance (AUC ≥ 0.80) across multiple matrices. In many studies, multivariate diagnostic panels were constructed using combinations of amino acids and other metabolites. As these panels did not consist exclusively of amino acids, they fall outside the scope of this review and are not further discussed.

## Discussion

This systematic review provides an overview of the literature on amino acid profiles associated with advanced colorectal neoplasia. Overall, no consistent advanced neoplasia-specific amino acid signature was identified within or across matrices. Most amino acids were reported as non-differential in the majority of studies, and significant results were often conflicting, with the exception of tissue, where a consistent trend towards elevated amino acid levels in CRC tissue was observed. Diagnostic performance of individual amino acids ranged widely (AUC 0.28–0.91), with the highest values originating from a faecal-based study. A key observation is the substantial methodological heterogeneity across studies, in both analytical techniques and data processing methods, which likely contributes to the observed variability. In addition, studies addressing early lesion detection remain scarce, highlighting a clear gap in the literature regarding the biomarker potential of amino acids in early stage disease.

Most included studies described the diagnostic performance of individual amino acids as part of broader metabolomics analyses. Only four out of twenty studies describing diagnostic performance employed external validation; in two of these, fewer than ten CRC patients were included, while the remaining two involved 30 and 59 patients with CRC. None of these studies reported an AUC exceeding 0.80 [18, 23, 25, 27]. Internal validation was performed in three studies [14, 28, 29], whereas no form of validation was applied in the remaining 13, limiting the generalisability of findings and increasing the risk of overestimating diagnostic performance. Moreover, three studies included larger CRC cohorts (up to 250 patients) but described poor diagnostic performance (AUC < 0.66) [30–32]. In addition to sample size and validation, the analytical approach (targeted vs. untargeted) also affects the robustness of findings. Half of the studies applied an untargeted approach without subsequent analytical confirmation of the amino acids using a targeted method, which may lead to uncertainty regarding the reliability of the identified metabolites. Furthermore, in 15 studies evaluating diagnostic biomarker panels, the best-performing models combined amino acids with other metabolites or proteins, outperforming amino acid–only panels. This may be explained by the fact that such panels capture a broader spectrum of tumour-specific metabolic alterations, reflecting coordinated changes across interconnected pathways, including amino acid metabolism, lipid synthesis, and host–microbiome interactions [1].

When examining consistency within individual biological matrices, CRC tissue showed a notably consistent pattern of increased amino acid concentrations across multiple studies. This finding aligns with the Warburg effect, the metabolic shift of tumour cells towards aerobic glycolysis, in which increased anabolic demands are supported by amino acids acting as carbon and nitrogen sources for biosynthesis [33]. Across studies, glutamate, glycine, and phenylalanine were most frequently elevated. Increased glutamate levels are consistent with enhanced glutamine dependency, a well-established feature of tumour metabolism, whereby glutamine is converted to glutamate and subsequently fuels the tricarboxylic acid (TCA) cycle and biosynthetic processes [33, 34]. Moreover, glutamate is strongly related to 2-oxoglutarate, the latter being an essential metabolite in the TCA cycle [35]. Elevated glycine aligns with increased one-carbon metabolism, which supplies one-carbon units for nucleotide synthesis and methylation reactions essential for DNA replication and cell division [36]. In addition, glycine and glutamate, together with cysteine, support glutathione synthesis and the detoxification of reactive oxygen species in tumour cells [36]. Phenylalanine elevation may reflect increased protein synthesis and turnover associated with rapid tumour growth, in addition to its involvement as a precursor in nonessential amino acid synthesis [36]. Together, these alterations highlight metabolic adaptations that support tumour proliferation and provide mechanistic insight into the consistent amino acid enrichment observed in CRC tissue.

In plasma, leucine and valine were consistently downregulated in all studies that assessed them, suggesting a potential role for these branched-chain amino acids in tumour-associated metabolic reprogramming. Leucine and valine are key substrates in energy production and protein synthesis, but are also involved in cell signalling pathways that regulate growth and proliferation, such as mTOR activation [37]. Their depletion may reflect increased uptake by proliferating cancer cells or altered systemic metabolism. Interestingly, despite the physiological similarity between plasma and serum, the amino acids reported as differentially abundant showed limited overlap, with serum studies more often reporting non-significant findings. This aligns with previous observations that serum generally contains higher and more variable amino acid concentrations [38].

The observed discrepancies in amino acid alterations within and between matrices may be explained by several underlying factors. First, tumour-specific characteristics, such as disease stage, tumour location, and age of onset, have been associated with variation in amino acid profiles, possibly due to altered gut microbiota composition [2, 39–42]. In addition, differences in amino acid profiles may arise from inherent biological variation between matrices. These include matrix-specific metabolic activity, differences in host–microbiota interactions, and variable amino acid stability or degradation during sample collection and processing [43–45]. Furthermore, although lifestyle-related factors, particularly diet, can significantly affect amino acid concentrations across sample types, most included studies rarely accounted for lifestyle variables [4, 46, 47]. While tumour-related and matrix-specific biological factors likely contribute to genuine variability in amino acid profiles, the inconsistent consideration of diet and variation in sample processing across studies suggests that a substantial proportion of the observed discrepancies may be methodological rather than biological in origin. Future studies should incorporate detailed dietary assessments and standardised sampling conditions (e.g. fasting versus non-fasting) to reduce this source of confounding.

Variation in analytical methods across studies likely represents an additional contributor to the inconsistencies observed in amino acid profiles. Although this review focused on the direction of amino acid alterations rather than absolute concentrations, differences in analytical sensitivity and dynamic range may still influence whether subtle group-level changes are detected. NMR, HPLC, and various mass spectrometry (MS) platforms were used, each differing in sensitivity, selectivity, and quantification accuracy. Although one study reported strong correlations between NMR and LC–MS/MS for most amino acids, systematic differences in absolute concentrations were observed, and group-level differences were not consistently detected across both methods [48]. HPLC remains a commonly used technique in amino acid analysis, yet coupling it to MS can substantially enhance sensitivity, especially in complex matrices such as faeces [49]. Even within MS-based approaches, methodological variation (e.g., GC–MS vs. LC–MS or CE–MS) may influence which amino acids are reliably quantified or detected [50, 51]. Variability in targeted versus untargeted approaches may further influence detection sensitivity and coverage [52], however, discrepancies in amino acid profiles were observed even among studies applying comparable analytical strategies. In addition, studies differed in their statistical approaches, including the use of univariate versus multivariate methods, correction for multiple testing, and criteria for reporting significance. Together, these methodological differences may contribute to the lack of consistency in amino acid profiles across studies.

A key strength of this review is its comprehensive scope, combining an extensive literature search with a structured comparison of amino acid profiles across multiple biological matrices. By evaluating findings from faeces, urine, serum, plasma, tissue and saliva side by side, this review provides a broad overview of matrix-specific trends and inconsistencies. In addition, diagnostic potential was assessed where applicable, offering clinically relevant insight beyond descriptive profiling.

### Limitations

This review has several limitations. First, a substantial methodological heterogeneity across studies, including differences in analytical techniques, statistical approaches, and study design, precluded formal meta-analysis and limited comparability. In addition, several included studies had small sample sizes, hampering power of their findings. Potential publication bias should also be considered. Untargeted metabolomics studies frequently reported only statistically significant results, while non-significant amino acids were often omitted. This selective reporting may have led to an overestimation of differential findings. To mitigate this, targeted and untargeted studies were analysed separately. Moreover, unaccounted heterogeneity related to pre-analytical factors, microbiota composition, and tumour characteristics may also have contributed to bias.

### Future perspectives

Future research should prioritise standardisation of analytical protocols to improve comparability between studies, as well as focus on early disease stages, particularly advanced adenomas and advanced serrated polyps. Identifying biomarkers that detect precancerous lesions remains critical for preventing their progression to CRC by endoscopic polypectomy. Importantly, rigorous validation strategies, including external validation, are essential to assess the true clinical utility of candidate biomarkers. Given that diagnostic performance often declines upon external validation, larger, well-characterised cohorts and harmonised methodologies will be key to advancing the field.

## Conclusion

In conclusion, individual amino acids do not appear to have sufficient diagnostic accuracy to serve as standalone biomarkers for CRC. Instead, amino acids profiling may be more informative when incorporated in multivariate panels that include other metabolite classes as well. Although tissue-based studies show the most consistent amino acid alterations, their limited suitability for non-invasive screening highlights the importance of translating these metabolic insights into clinically applicable biomarker panels.

## Supplementary Information

Below is the link to the electronic supplementary material.

Supplementary file1 (DOCX 321 KB)

Supplementary file2 (XLSX 82 KB)

## Data Availability

The primary data supporting the findings of this study are included in the article and provided in Appendix B. Additional data may be obtained from the corresponding author upon reasonable request.
