# Supplementary material for: Colorectal neoplasia-specific amino acid profiles and their diagnostic potential: a systematic review
Source: Clin Transl Oncol. 2026 Mar 28;28(9):4042–60. doi: 10.1007/s12094-026-04278-9 (PMC13499740; doi:10.1007/s12094-026-04278-9)
Supplement: Supplementary file 1 [file 12094_2026_4278_MOESM1_ESM.docx]

**Appendix A. Supplementary information**

Colorectal neoplasia specific amino-acid profiles and their diagnostic potential: a systematic review

Roza. C.M. Opperman^a,b,c^, Sofie. Bosch^a,b^, Eduard A. Struys^d^, Awa. Hassan^a^, Faridi S. Jamaludin^e^, Tim G.J. de Meij^b,f,g^, Evelien Dekker^c,h^, Nanne K.H. de Boer^a,b^

^a^Department of Gastroenterology and Hepatology, Amsterdam UMC, Vrije Universiteit Amsterdam, 1081 HV Amsterdam, The Netherlands.

^b^Amsterdam Gastroenterology Endocrinology Metabolism (AGEM) Research Institute, 1081 HV Amsterdam, The Netherlands.

^c^Cancer Center Amsterdam, research program, 1081 HV Amsterdam, The Netherlands.

^d^Department of Laboratory Medicine, Amsterdam University Medical Centre, 1105 AZ Amsterdam, The Netherlands.

^e^Amsterdam UMC location University of Amsterdam, Medical Library AMC, Meibergdreef 9, 1105 AZ Amsterdam, the Netherlands.

^f^Department of Paediatric Gastroenterology, Emma Children's Hospital, Amsterdam UMC, Vrije Universiteit Amsterdam, 1081 HV Amsterdam, The Netherlands.

^g^Department of Paediatric Gastroenterology, Emma Children's Hospital, Amsterdam UMC, Academic Medical Centre, 1105 AZ Amsterdam, The Netherlands.

^h^Department of Gastroenterology and Hepatology, Amsterdam UMC, University of Amsterdam, 1081 HV Amsterdam, the Netherlands.

**Correspondence address:** R.C.M. Opperman, MD. Department of Gastroenterology and Hepatology, Amsterdam UMC, Vrije Universiteit Amsterdam, 1081 HV Amsterdam, The Netherlands. ORCiD: 0000-0002-7279-0940 E-mail: [r.opperman@amsterdamumc.nl](mailto:r.opperman@amsterdamumc.nl)

**Table of content**

| **Topic** | **Page** |
| --- | --- |
| **Supplementary Text 1.** The Newcastle-Ottawa Scale (NOS) for assessing the quality of included case-control studies. | 3 |
| **Supplementary Text 2.** The Newcastle-Ottawa Scale (NOS) for assessing the quality of included cross-sectional studies. | 4 |
| **Supplementary figure 1**. Directional changes in amino acid concentrations for colorectal cancer versus controls, stratified by analytical approach. | 5 |
| **Supplementary Table 1**. Detailed overview of the search strategy. | 6 |
| **Supplementary Table 2.** List of included amino acids. | 8 |
| **Supplementary Table 3.** Distribution of primary analytical platforms across biological matrices and targeted versus untargeted studies. | 9 |
| **Supplementary Table 4**. Extracted study data for the colorectal carcinoma versus control comparison. | Appendix B |
| **Supplementary Table 5**. Extracted study data for the advanced adenoma versus control comparison. | Appendix B |
| **Supplementary Table 6**. Extracted study data for the advanced neoplasia versus control comparison. | Appendix B |
| **Supplementary Table 7.** Quality assessment of included case-control studies according to the Newcastle-Ottawa Scale. | 10 |
| **Supplementary Table 8.** Quality assessment of included cross sectional studies according to the modified Newcastle-Ottawa Scale *(Modesti et al., 2016).* | 13 |
| **Supplementary Table 9**. Overview of diagnostic performance for individual amino acids. | 14 |

**Supplementary Text 1.** The Newcastle-Ottawa Scale (NOS) for assessing the quality of included case-control studies.

SELECTION

1) Is the Case Definition Adequate?
   a. Requires some independent validation (e.g. >1 person/record/time/process to extract information, or reference to primary record source such as x-rays or medical/hospital records) ☆
   b. Record linkage (e.g. ICD codes in database) or self-report with no reference to primary record
   c. No description
2) Representativeness of the Cases
   a. All eligible cases with outcome of interest over a defined period of time, all cases in a defined catchment area, all cases in a defined hospital or clinic, group of hospitals, health maintenance organisation, or an appropriate sample of those cases (e.g. random sample) ☆
   b. Not satisfying requirements in part (a), or not stated.
3) Selection of Controls

This item assesses whether the control series used in the study is derived from the same population as the cases and essentially would have been cases had the outcome been present.

a. Community controls (i.e. same community as cases and would be cases if had outcome) ☆
b. Hospital controls, within same community as cases (i.e. not another city) but derived from a hospitalised population
c. No description

4) Definition of Controls
   a. If cases are first occurrence of outcome, then it must explicitly state that controls have no history of this outcome. If cases have new (not necessarily first) occurrence of outcome, then controls with previous occurrences of outcome of interest should not be excluded. ☆
   b. No mention of history of outcome

COMPARABILITY

1) Comparability of Cases and Controls on the Basis of the Design or Analysis

A maximum of 2 stars can be allotted in this category

Either cases and controls must be matched in the design and/or confounders must be adjusted for in the analysis. Statements of no differences between groups or that differences were not statistically significant are not sufficient for establishing comparability. Note: If the odds ratio for the exposure of interest is adjusted for the confounders listed, then the groups will be considered to be comparable on each variable used in the adjustment.

There may be multiple ratings for this item for different categories of exposure (e.g. ever vs. never, current vs. previous or never)

Age = ☆ , Other controlled factors = ☆

EXPOSURE

1) Ascertainment of Exposure

Allocation of stars as per rating sheet

2) Same method of ascertainment for cases and controls

Allocation of stars in case of yes

2) Non-Response Rate

Allocation of stars as per rating sheet

**Supplementary Text 2.** The Newcastle-Ottawa Scale (NOS) for assessing the quality of included cross-sectional studies.

*Based on the modified Newcastle-Ottawa Scale (Modesti et al., 2016)*

Selection: (Maximum 5 stars)

1) Representativeness of the sample:
   a. Truly representative of the average in the target population. ☆ (all subjects or random sampling)
   b. Somewhat representative of the average in the target population. ☆ (non-random sampling)
   c. Selected group of users.
   d. No description of the sampling strategy.
2) Sample size:
   a. Justified and satisfactory. ☆
   b. Not justified.
3) Non-respondents:
   a. Comparability between respondents and non-respondents characteristics is established, and the response rate is satisfactory. ☆
   b. The response rate is unsatisfactory, or the comparability between respondents and non-respondents is unsatisfactory.
   c. No description of the response rate or the characteristics of the responders and the non-responders.
4) Ascertainment of the exposure (risk factor):
   a. Validated measurement tool. ☆☆
   b. Non-validated measurement tool, but the tool is available or described. ☆
   c. No description of the measurement tool.

Comparability: (Maximum 2 stars)

1) The subjects in different outcome groups are comparable, based on the study design or analysis. Confounding factors are controlled.
   a. The study controls for the most important factor (select one). ☆
   b. The study control for any additional factor. ☆

Outcome: (Maximum 3 stars)

1) Assessment of the outcome:
   a. Independent blind assessment. ☆☆
   b. Record linkage. ☆☆
   c. Self report. ☆
   d. No description.
2) Statistical test:
   a. The statistical test used to analyse the data is clearly described and appropriate, and the measurement of the association is presented, including confidence intervals and the probability level (p value). ☆
   b. The statistical test is not appropriate, not described or incomplete.

**Supplementary Figure 1**. Directional changes in amino acid concentrations for colorectal cancer versus controls, stratified by analytical approach.

Panel a displays findings from targeted studies; panel b shows results from untargeted studies. For each approach, a matrix-specific overview is provided for the 20 amino acids included in this review. The Y-axis lists the amino acids; the X-axis indicates the biological matrices. Each circle reflects the number of studies reporting a significant increase (red), decrease (blue), or no significant difference (ND, grey) in amino acid levels. Circle size corresponds to the number of studies, with larger circles indicating greater consistency. Numbers within the circles denote how many studies reported the respective outcome out of the total number of studies assessing that amino acid in that matrix. Deng et al. (2024) reported L- and D-enantiomers separately; only amino acids showing significant changes in both forms were included for consistency.

**Supplementary Table 1**. Detailed overview of the search strategy

| **#** | **Search** |
| --- | --- |
| **MEDLINE (via Ovid):**  Database(s): Ovid MEDLINE(R) ALL 1946 to May 21, 2025 | |
| 1 | biomarkers/ or biomarkers, tumor/ or Microbiota/ |
| 2 | (marker* or biomarker* or bio-marker*).ti,ab,kf. |
| 3 | ((fecal or faecal or feces or stool or gut or saliva or tissue or urine) adj3 (microbiota* or microbiom*)).ti,ab,kf. |
| 4 | 1 or 2 or 3 |
| 5 | exp colorectal neoplasms/ or exp rectal neoplasms/ or Colonic Polyps/ or exp Cecal Neoplasms/ |
| 6 | ((colo* or rectum* or rectal* or sigmoid* or cecal or cecum or caecal or caecum) adj3 (neoplasm* or cancer* or tumo* or adeno* or carcino* or metastas* or polyp*)).ti,ab,kf. |
| 7 | (high risk polyp* or advanced adenoma* or serrated polyp* or hyperplastic polyp* or sessile serrated lesion* or traditional serrated adenoma*).ti,ab,kf. |
| 8 | 5 or 6 or 7 |
| 9 | exp Amino Acids/ or exp Metabolomics/ or exp Metabolome/ |
| 10 | (amino acid* or metabolic* or metabolite* or metabonomic* or metabolome* or metabolomic* or alanine or arginine or aspartic acid or aspargine or cysteine or glutamine or Glutamic acid or glycine or histidine or isoleucine or leucine or lysine or methionine or phenylalanine or proline or serine or threonine or tyrosine or tryptophan or valine).ti,ab,kf. |
| 11 | 9 or 10 |
| 12 | 4 and 8 and 11 |
| 13 | ((exp Animals/ or exp Animal Experimentation/ or exp models, animal/ or (animal* or monkey* or sheep or ovine or lamb or lambs or goat* or pig or pigs or swine or porcine or pup or pups or dog or dogs or canine or bitch* or beagle or feline or rodent* or rabbit* or rat or rats or mice or mouse or murine or cow or cows or horse or horses or ape or apes or gorilla or gorillas).ti,ab,kw.) not Humans/) or animal*.ti. |
| 14 | exp child/ not (adult/ and aged/ and middle aged/) |
| 15 | exp Pediatrics/ or (pediatr* or paediatr* or peadiatr*).ti. |
| 16 | 14 or 15 |
| 17 | 12 not 13 not 16 |
| 18 | comment/ or editorial/ or exp "review"/ or "systematic review"/ or (comment* or editorial or systematic review).ti. |
| 19 | 17 not 18 |
| **EMBASE (via Ovid):**  Database(s): Embase Classic+Embase 1947 to 2025 May 21 | |
| 1 | marker/ or biochemical marker/ or biological marker/ or environmental marker/ or molecular marker/ or pharmacological biomarker/ or exp tumor marker/ or microbiome/ |
| 2 | (marker* or biomarker* or bio-marker*).ti,ab,kf. |
| 3 | ((fecal or faecal or feces or stool or gut or saliva or tissue or urine) adj3 (microbiota* or microbiom*)).ti,ab,kf. |
| 4 | 1 or 2 or 3 |
| 5 | exp colon tumor/ or exp rectum tumor/ or exp colon polyp/ or colon adenoma/ or colorectal adenoma/ or rectum adenoma/ or exp colon adenoma/ or exp colorectal adenoma/ or exp cecum tumor/ |
| 6 | ((colo* or rectum* or rectal* or sigmoid* or cecal or cecum or caecal or caecum) adj3 (neoplasm* or cancer* or tumo* or adeno* or carcino* or metastas* or polyp*)).ti,ab,kf. |
| 7 | (high risk polyp* or advanced adenoma* or serrated polyp* or hyperplastic polyp* or sessile serrated lesion* or traditional serrated adenoma*).ti,ab,kf. |
| 8 | 5 or 6 or 7 |
| 9 | exp amino acid/ or exp metabolomics/ or metabolome/ |
| 10 | (amino acid* or metabolic* or metabolite* or metabonomic* or metabolome* or metabolomic* or alanine or arginine or aspartic acid or aspargine or cysteine or glutamine or Glutamic acid or glycine or histidine or isoleucine or leucine or lysine or methionine or phenylalanine or proline or serine or threonine or tyrosine or tryptophan or valine).ti,ab,kf. |
| 11 | 9 or 10 |
| 12 | 4 and 8 and 11 |
| 13 | ((exp animal/ or exp animal experiment/ or exp animal model/ or exp veterinary medicine/ or (animal* or monkey* or sheep or ovine or lamb or lambs or goat* or pig or pigs or swine or porcine or pup or pups or dog or dogs or canine or bitch* or beagle or feline or rodent* or rabbit* or rat or rats or mice or mouse or murine or cow or cows or horse or horses or ape or apes or gorilla or gorillas).ti,ab,kw.) not human/) or animal*.ti. |
| 14 | exp child/ not (adult/ or exp aged/ or middle aged/) |
| 15 | exp pediatrics/ or (pediatr* or paediatr* or peadiatr*).ti. |
| 16 | 14 or 15 |
| 17 | 12 not 13 not 16 |
| 18 | editorial/ or note/ or "review"/ or "systematic review"/ or (comment* or editorial or systematic review).ti. |
| 19 | 17 not 18 |
| 20 | limit 19 to conference abstract status |
| 21 | 19 not 20 |
| **Cochrane Central Register of Controlled Trials**  Issue 4 of 12, April 2025 | |
| 1 | (marker* or biomarker* or bio-marker*):ti,ab,kw |
| 2 | ((fecal or faecal or feces or stool or gut or saliva or tissue or urine) near/3 (microbiota* or microbiom*)):ti,ab,kw |
| 3 | #1 or #2 |
| 4 | ((colo* or rectum* or rectal* or sigmoid* or cecal or cecum or caecal or caecum) near/3 (neoplasm* or cancer* or tumo* or adeno* or carcino* or metastas* or polyp*)):ti,ab,kw |
| 5 | (high risk polyp* or advanced adenoma* or serrated polyp* or hyperplastic polyp* or sessile serrated lesion* or traditional serrated adenoma*):ti,ab,kw |
| 6 | #4 or #5 |
| 7 | (amino acid* or metabolic* or metabolite* or metabonomic* or metabolome* or metabolomic* or alanine or arginine or aspartic acid or aspargine or cysteine or glutamine or Glutamic acid or glycine or histidine or isoleucine or leucine or lysine or methionine or phenylalanine or proline or serine or threonine or tyrosine or tryptophan or valine):ti,ab,kw |
| 8 | #3 and #6 and #7 in Trials |

**Supplementary Table 2.** List of included amino acids

| Amino acid | |
| --- | --- |
| 1. | Alanine |
| 2. | Arginine |
| 3. | Aspartic acid |
| 4. | Asparagine |
| 5. | Cysteine |
| 6. | Glutamine |
| 7. | Glutamate |
| 8. | Glycine |
| 9. | Histidine |
| 10. | Isoleucine |
| 11. | Leucine |
| 12. | Lysine |
| 13. | Methionine |
| 14. | Phenylalanine |
| 15. | Proline |
| 16. | Serine |
| 17. | Threonine |
| 18. | Tyrosine |
| 19. | Tryptophan |
| 20. | Valine |

**Supplementary Table 3.** Distribution of primary analytical platforms across biological matrices and targeted versus untargeted studies.**^a^**

|  | Primary analytical methods per matrix (targeted / untargeted) | | | | | Overall use of targeted and untargeted approaches per analytical platform | |
| --- | --- | --- | --- | --- | --- | --- | --- |
|  | **Faeces** | **Serum** | **Plasma** | **Urine** | **Tissue** | **Targeted** | **Untargeted** |
| **NMR** | 4 (0/4) | 3 (0/3) | - | 1 (0/1) | 8 (0/8) | - | 16 |
| **HPLC** | 1 (1/0) | 1 (1/0) | - | - | - | 2 |  |
| **CE-MS** | 1 (1/0) | 1 (0/1) | - | 1 (0/1) | 1 (0/1) | 1 | 3 |
| **GC-MS** | 4 (2/2) | 7 (3/4) | 2 (2/0) | - | 7 (4/4)^b^ | 11 | 10^b^ |
| **LC-MS** | 7 (2/5) | 8 (5/3) | 5 (3/2) | 4 (3/1) | 7 (1/6) | 14 | 17 |
| **FIA-MS** | - | 2 (2/0) | - | - | - | 2 | - |
| **TOF-SIMS** | - | - | - | - | 1 (0/1) | - | 1 |

^a^ Values represent the number of studies applying each analytical platform, with parentheses indicating the number of targeted and untargeted studies, respectively. Abbreviations: CE-MS – Capillary Electrophoresis–Mass Spectrometry; FIA-MS/MS – Flow Injection; Analysis–Tandem Mass Spectrometry; GC-MS – Gas Chromatography–Mass Spectrometry; HPLC - High-Performance Liquid Chromatography; LC-MS – Liquid Chromatography–Mass Spectrometry; NMR spectroscopy – Nuclear Magnetic Resonance Spectroscopy; TOF-SIMS – Time-of-Flight Secondary Ion Mass Spectrometry.

^b^ one study performed untargeted for discovery and targeted for validation

**Supplementary Table 7.** Quality assessment of included case-control studies according to the Newcastle-Ottawa Scale

|  | **Selection** | | | | **Comparability** | | **Exposure** | | | **Score** |
| --- | --- | --- | --- | --- | --- | --- | --- | --- | --- | --- |
| **First author [publication year]** | Is the case  definition  adequate? | Representativeness  of the cases | Selection  of controls | Definition  of controls | Comparability  Of baseline characteristic: age | Comparability  Of baseline characteristic: other | Ascertainment  of exposure | Same method for cases and controls | Nonresponse  rate |  |
| Arima [2020] | ☆ | ☆ | ☆^a^ | ☆ | ☆ | ☆, paired design | ☆ | ☆ | - | 8/9 |
| Bednarz-Misa [2020] | ☆^b^ | ☆ | ☆ | -^c^ | ☆ | ☆, sex | ☆ | ☆ | - | 7/9 |
| Bosch [2022] | ☆ | ☆ | ☆ | ☆ | ☆ | ☆, BMI and smoking status | ☆ | ☆ | - | 8/9 |
| Cai [2020] | ☆ | ☆ | - | ☆ | ☆ | - | ☆ | ☆ | - | 6/9 |
| Cai [2025] | ☆ | ☆ | ☆^a^ | ☆ | ☆ | ☆, paired design | ☆ | ☆ | - | 8/9 |
| Chan [2009] | ☆ | ☆ | ☆^a^ | ☆ | ☆ | ☆, paired design | ☆ | ☆ | - | 8/9 |
| Cho [2021] | ☆ | ☆ | ☆ | -^c^ | ☆ | ☆, paired design | ☆ | ☆ | - | 7/9 |
| Coradduzza [2022] | ☆ | ☆ | ☆ | -^c^ | - | - | ☆ | ☆ | - | 5/9 |
| Deng [2019] | ☆ | ☆ | ☆ | ☆ | - | ☆, sex | ☆ | ☆ | - | 7/9 |
| Deng [2020] | ☆ | ☆ | ☆ | -^c^ | ☆ | ☆, sex | ☆ | ☆ | - | 7/9 |
| Deng [2024] | ☆ | ☆ | ☆ | -^c^ | - | - | ☆ | ☆ | - | 5/9 |
| Denkert [2008] | ☆ | ☆ | ☆^a^ | ☆ | ☆ | ☆, paired design | ☆ | ☆ | - | 8/9 |
| Du [2022] | ☆ | ☆ | ☆ | ☆ | ☆ | ☆, sex | ☆ | ☆ | - | 8/9 |
| Eylem [2020] | ☆ | ☆ | ☆^a^ | ☆ | - | - | ☆ | ☆ | - | 6/9 |
| Farshidfar [2016] | ☆ | ☆ | ☆ | ☆ | ☆ | ☆, sex | ☆ | ☆ | - | 8/9 |
| Farshidfar [2018] | ☆^b^ | ☆ | ☆ | ☆ | ☆ | ☆, sex | ☆ | ☆ | - | 8/9 |
| Faizi [2025] | ☆^b^ | ☆ | ☆ | ☆ | - | - | ☆ | ☆ | - | 6/9 |
| Le Gall [2018] | ☆ | ☆ | ☆ | ☆ | ☆ | ☆, sex | ☆ | ☆ | - | 8/9 |
| Gao [2016] | ☆ | ☆ | ☆^a^ | ☆ | ☆ | ☆, paired design | ☆ | ☆ | - | 8/9 |
| Geijsen [2019] | ☆ | ☆ | ☆ | ☆ | ☆ | ☆, sex, BMI, smoking status | ☆ | ☆ | - | 8/9 |
| Gu [2019] | ☆^b^ | ☆ | ☆ | -^c^ | - | - | ☆ | ☆ | - | 5/9 |
| Guo [2023] | ☆^b^ | ☆ | ☆ | ☆ | ☆ | ☆, sex | ☆ | ☆ | - | 8/9 |
| Huang [2002] | ☆ | ☆ | ☆ | -^c^ | - | - | ☆ | ☆ | - | 5/9 |
| Ikeda [2011] | ☆ | ☆ | ☆ | -^c^ | - | - | ☆ | ☆ | - | 5/9 |
| Jiménez [2013] | ☆ | ☆ | ☆^a^ | ☆ | ☆ | ☆, paired design | ☆ | ☆ | - | 8/9 |
| Kim, D. [2020] | ☆ | ☆ | ☆ | -^c^ | - | - | ☆ | ☆ | - | 5/8 |
| Kim, W. [2020] | ☆ | ☆ | ☆ | ☆ | ☆ | ☆, sex, race, smoking status. | ☆ | ☆ | - | 8/9 |
| Kim [2019] | ☆ | ☆ | ☆ | ☆ | - | - | ☆ | ☆ | - | 6/9 |
| Kinross [2017] | ☆ | ☆ | ☆^a^ | ☆ | ☆ | ☆, paired design | ☆ | ☆ | - | 8/9 |
| Kong [2023] | ☆ | ☆ | ☆ | ☆ | ☆ | ☆, sex | ☆ | ☆ | - | 8/9 |
| Kulecka [2024] | ☆ | ☆ | ☆ | -^c^ | ☆ | ☆, sex | ☆ | ☆ | - | 7/9 |
| Leichtle [2012] | ☆ | ☆ | ☆ | -^c^ | ☆ | ☆, sex | ☆ | ☆ | - | 7/9 |
| Li [2019] | ☆ | ☆ | ☆ | -^c^ | ☆ | ☆, sex | ☆ | ☆ | - | 7/9 |
| Li [2019] | ☆ | ☆ | ☆ | -^c^ | ☆ | ☆, sex, BMI | ☆ | ☆ | - | 7/9 |
| Liang [2024] | ☆ | ☆ | ☆ | ☆ | ☆ | ☆, sex | ☆ | ☆ | - | 8/9 |
| Lin [2016] | ☆ | ☆ | ☆ | -^c^ | - | - | ☆ | ☆ | - | 5/8 |
| Long [2017] | ☆^b^ | ☆ | ☆ | -^c^ | ☆ | ☆, sex, ethnicity, residence | ☆ | ☆ | - | 7/9 |
| Long [2020] | ☆ | ☆ | ☆^a^ | ☆ | ☆ | ☆, paired design | ☆ | ☆ | - | 8/9 |
| Lv [2020] | ☆ | ☆ | ☆^a^ | ☆ | ☆ | ☆, paired design | ☆ | ☆ | - | 8/9 |
| Mal [2012] | ☆ | ☆ | ☆^a^ | ☆ | ☆ | ☆, paired design | ☆ | ☆ | - | 8/9 |
| Manna [2014] | ☆ | ☆ | ☆^a^ | ☆ | ☆ | ☆, paired design | ☆ | ☆ | - | 8/9 |
| Miyagi [2011] | ☆ | ☆ | ☆ | -^c^ | ☆ | ☆, sex | ☆ | ☆ | - | 7/9 |
| Monleón [2008] | ☆ | ☆ | ☆ | ☆ | - | - | ☆ | ☆ | - | 6/9 |
| Moreno [1996] | ☆ | ☆ | ☆^a^ | ☆ | ☆^d^ | - | ☆ | ☆ | - | 7/9 |
| Ning [2017] | ☆ | ☆ | ☆^a^ | ☆ | ☆ | ☆, paired design | ☆ | ☆ | - | 8/9 |
| Ning [2021] | ☆ | ☆ | ☆ | -^c^ | - | - | ☆ | ☆ | - | 5/9 |
| Nishiumi [2012] | ☆ | ☆ | ☆ | -^c^ | ☆ | ☆, sex | ☆ | ☆ | - | 7/9 |
| Nishiumi [2017] | ☆ | ☆ | ☆ | ☆ | ☆ | ☆, sex | ☆ | ☆ | - | 8/9 |
| Okamoto [2008] | ☆ | ☆ | ☆ | -^c^ | ☆ | ☆, sex | ☆ | ☆ | - | 7/9 |
| Phua [2014] | ☆ | ☆ | ☆ | ☆ | ☆^e^ | - | ☆ | ☆ | - | 7/9 |
| Qiu [2009] | ☆ | ☆ | ☆ | -^c^ | - | - | ☆ | ☆ | - | 5/9 |
| Qiu [2014] | ☆ | ☆ | ☆^a^ | ☆ | ☆^d^ | - | ☆ | ☆ | - | 7/9 |
| Ramzy [2025] | ☆ | ☆ | ☆ | ☆ | ☆ | ☆, sex | ☆ | ☆ | - | 8/9 |
| Rao [2024] | ☆ | ☆ | ☆^a^ | ☆ | ☆ | ☆, paired design | ☆ | ☆ | - | 8/9 |
| Rodriguez-Tomas [2021] | ☆ | ☆ | ☆ | -^c^ | ☆ | - | ☆ | ☆ | - | 6/9 |
| Su [2025] | ☆ | ☆ | ☆ | ☆ | - | - | ☆ | ☆ | - | 6/9 |
| Sun [2020] | ☆^b^ | ☆ | ☆ | ☆ | ☆ | ☆, sex, BMI | ☆ | ☆ | - | 8/9 |
| Sun [2024] | ☆^b^ | ☆ | ☆ | ☆ | - | - | ☆ | ☆ | - | 6/9 |
| Tan [2013] | ☆ | ☆ | ☆ | -^c^ | ☆ | ☆, sex | ☆ | ☆ | - | 7/9 |
| Telleria [2022] | ☆ | ☆ | ☆ | ☆ | - | ☆, sex | ☆ | ☆ | - | 7/9 |
| Tevini [2022] | ☆ | ☆ | ☆ | ☆ | - | - | ☆ | ☆ | - | 6/9 |
| Tristán [2023] | ☆ | ☆ | ☆ | -^c^ | ☆ | ☆, sex | ☆ | ☆ | - | 7/9 |
| Uchiyama [2017] | ☆^b^ | ☆ | ☆ | ☆ | ☆ | ☆, sex | ☆ | ☆ | - | 8/9 |
| Wang [2023] | ☆ | ☆ | ☆^a^ | ☆ | - | - | ☆ | ☆ | - | 6/9 |
| Wang [2017] | ☆ | ☆ | ☆ | -^c^ | ☆ | ☆, sex | ☆ | ☆ | - | 7/9 |
| Wu [2020] | ☆ | ☆ | ☆ | -^c^ | ☆ | ☆, sex | ☆ | ☆ | - | 7/9 |
| Yagin, F. [2023] | ☆ | ☆ | ☆ | -^c^ | - | - | ☆ | ☆ | - | 5/8 |
| Yang [2019] | ☆ | ☆ | ☆ | -^c^ | - | - | ☆ | ☆ | - | 5/9 |
| Wang [2020] | ☆ | ☆ | ☆^a^ | ☆ | ☆ | ☆, paired design | ☆ | ☆ | - | 8/9 |
| Zha [2018] | ☆ | ☆ | ☆^a^ | ☆ | ☆ | ☆, paired design | ☆ | ☆ | - | 8/9 |

^a^Although controls were derived from the same patients (paired normal tissue), this ensures tight biological comparability and minimizes inter-individual variation. Therefore, one star was granted.

^b^Colorectal cancer adequately described and diagnosed with colonoscopy, polyp adequately diagnosed with colonoscopy but study does not provide a detailed histological definition or subclassification (e.g., tubular, villous, dysplasia: advanced/non-advanced). Therefore, in this review, comparisons involving the polyp group have been excluded.

^c^Not diagnosed with golden standard colonoscopy but sufficient enough to include in the systematic review.

^d^Although part of the analysis used a paired design, another comparison was based on more tumour tissue versus partly matched normal mucosa samples, without matching or adjustment for confounding factors; therefore, one star was granted.

^e^Tissue analysis was paired (tumour and normal mucosa from the same patient), which controls for intra-individual variation. However, faecal comparisons were unpaired and controls were randomly selected without matching or adjustment for potential confounders such as age or sex. Therefore, one star was granted.

**Supplementary Table 8.** Quality assessment of included cross sectional studies according to the modified Newcastle-Ottawa Scale *(Modesti et al., 2016)*

|  | **Selection** | | | | **Comparability** | | **Outcome** | | **Score** |
| --- | --- | --- | --- | --- | --- | --- | --- | --- | --- |
| **First author [publication year]** | Representativeness of sample | Sample size | Non-respondents | Ascertainment of the exposure (risk factor) | Comparability  Of baseline characteristic: age | Comparability  Of baseline characteristic: Other | Assessment of outcome | Statistical test |  |
| Coker [2022] | ☆^a^ | ☆ | - | ☆☆ | ☆ | ☆, sex, obesity | ☆☆ | ☆ | 9/10 |
| Deng [2016] | ☆^a^ | ☆ | - | ☆☆ | - | - | ☆☆ | ☆ | 7/10 |
| Erben [2021] | ☆ | ☆ | - | ☆☆ | - | ☆, sex | ☆☆ | ☆ | 8/10 |
| Yachida [2019] | ☆ | ☆ | - | ☆☆ | - | - | ☆☆ | ☆ | 7/10 |
| Zhang [2023] | ☆^a^ | ☆ | - | ☆☆ | ☆ | ☆, multiple factors | ☆☆ | ☆ | 9/10 |
| Zhang [2024] | ☆ | ☆ | - | ☆☆ | ☆ | ☆, sex | ☆☆ | ☆ | 9/10 |
| Zhu [2014] | ☆^a^ | ☆ | - | ☆☆ | ☆ | ☆, sex | ☆☆ | ☆ | 9/10 |

^a^ Colorectal cancer adequately described and diagnosed with colonoscopy, polyp adequately diagnosed with colonoscopy but study does not provide a detailed histological definition or subclassification (e.g., tubular, villous, dysplasia: advanced/non-advanced). Therefore, in this review, comparisons involving the polyp group have been excluded.

**Supplementary Table 9.** Diagnostic performance of individual amino acids across matrices

| **Author [year]** | **Matrix** | **Comparison** | **Validation strategy** | **Amino acid** | **AUC (95%CI)** | **Sensitivity (%)** | **Specificity (%)** | |
| --- | --- | --- | --- | --- | --- | --- | --- | --- |
| Bosch, S. [2022] | Faeces | CRC (n=12) vs. controls (n=20) | 100× bootstrapping for feature selection; AUC distribution compared to random label sampling. | Proline | 0.718 |  | |  |
| Du, X. [2022] | Faeces | CRC (n=30) vs. controls (n=33) | Logistic regression model with leave-one-out cross-validation. | Tryptophan | 0.652 |  |  | |
| Lin, Y. [2019] | Faeces | CRC (n=50) vs. controls (n=50) | ROC analyses, no validation described for individual amino acids. | Glutamate | 0.595 (0.447–0.732) |  |  | |
|  |  |  |  | Alanine | 0.602 (0.453–0.737) |  |  |  |
| Lin, Y. [2016] | Faeces | CRC stage I/II (n=20) vs. controls (n=32) | ROC analyses, no validation described for individual amino acids. | Glutamine | 0.908 (0.795-1.022) | 92 | 77 | |
|  |  |  |  | Proline | 0.845 (0.676-0.993) | 74 | 92 | |
|  |  |  |  | Alanine | 0.864 (0.712-1.016) | 77 | 99 | |
|  |  |  |  | Valine | 0.840 (0.692-0.962) | 100 | 47 | |
|  |  |  |  | Leucine | 0.840 (0.692-0.962) | 100 | 47 | |
|  |  |  |  | Isoleucine | 0.840 (0.692-0.962) | 100 | 47 | |
|  |  |  |  | Glutamate | 0.849 (0.693-1.006) | 62 | 85 | |
| Yagin, F. [2023] | MEV | CRC (n=36) vs. controls (n=40) | ROC analyses, no validation described for individual amino acids. | Alanine | 0.601(0.471–0.725) | 43 | 81 | |
|  |  |  |  | Isoleucine | 0.783(0.679–0.875) | 75 | 67 | |
|  |  |  |  | Leucine | 0.765(0.646–0.861) | 82 | 64 | |
|  |  |  |  | Lysine | 0.556(0.435–0.687) | 60 | 61 | |
| Deng. W. [2024] | Urine | CRC (n=155) vs. controls (n=115) | ROC analyses, no validation described for individual amino acids. | L-Alanine | 0.807(0.756-0.858) | 69 | 80 | |
|  |  |  |  | D-Alanine | 0.728(0.666-0.789) | 46 | 90 | |
|  |  |  |  | D-Glutamine | 0.626(0.556-0.696) | 39 | 87 | |
|  |  |  |  | D-Serine | 0.708(0.646-0.770) | 58 | 75 | |
| Wang, Z. [2017] | Urine | Validation set: CRC (n=10) vs. controls (n=8) | Markers selected on training set (45 CRC and 32 controls). | Phenylalanine | 0.772 (0.642-0.902) | 49 | 100 | |
|  |  |  |  | Asparagine | 0.763 (0.628-0.898) | 70 | 75 | |
|  |  |  |  | Cysteine | 0.792 (0.672-0.913) | 57 | 90 | |
|  |  |  |  | Alanine | 0.761 (0.628-0.894) | 70 | 72 | |
|  |  |  |  | Glutamine | 0.722(0.587-0.857) | 75 | 65 | |
| Bednarz-Misa, I. [2020] | Serum | CRC (n=137) vs. controls (n=54) | ROC analyses, no validation described for individual amino acids. | Arginine | 0.519 (0.45-0.59) | 40 | 83 | |
| Ikeda, A. [2012] | Serum | CRC (n=12) vs. controls (n=12) | ROC analyses, no validation described for individual amino acids. | Alanine | 0.656 (0.634-0.948) | 55 | 92 | |
|  |  |  |  | Glutamine | 0.758 (0.727-1.007) | 82 | 67 | |
| Leichtle, A. [2012] | Serum | CRC (n=59) vs. controls (n=58) | Logistic regression with Bayesian model averaging (BMA), validated using bootstrapping (B = 999) and correlation-adjusted marginal correlations (CAR). | Alanine | 0.677 (0.580-0.774) |  |  | |
|  |  |  |  | Arginine | 0.594 (0.490-0.679) |  |  | |
|  |  |  |  | Aspartic acid | 0.622 (0.520-0.724) |  |  | |
|  |  |  |  | Glutamine | 0.530 (0.424-0.653) |  |  | |
|  |  |  |  | Glycine | 0.707 (0.613-0.801) |  |  | |
|  |  |  |  | Histidine | 0.691 (0.593-0.788) |  |  | |
|  |  |  |  | Leucine/Isoleucine | 0.653 (0.551-0.754) |  |  | |
|  |  |  |  | Lysine | 0.624 (0.522-0.725) |  |  | |
|  |  |  |  | Methionine | 0.633 (0.532-0.735) |  |  | |
|  |  |  |  | Proline | 0.463 (0.357-0.569) |  |  | |
|  |  |  |  | Serine | 0.506 (0.399-0.612) |  |  | |
|  |  |  |  | Threonine | 0.645 (0.544-0.746) |  |  | |
|  |  |  |  | Tryptophan | 0.506 (0.399-0.612) |  |  | |
|  |  |  |  | Tyrosine | 0.636 (0.5360.737) |  |  | |
|  |  |  |  | Valine | 0.625 (0.521-0.728) |  |  | |
| Nishiumi, S. [2012] | Serum | Training: CRC (n=60) vs. controls (n=60) | Logistic regression with stepwise variable selection. | Isoleucine | 0.672 (0.570–0.760) | 77 | 53 | |
|  |  |  |  | Aspartic acid | 0.700 (0.596–0.787) | 65 | 80 | |
|  |  |  |  | Glutamate | 0.755 (0.656–0.833) | 77 | 67 | |
|  |  |  |  | Asparagine | 0.605 (0.500–0.702) | 67 | 55 | |
|  |  | Validation: CRC (n=59) vs. controls (n=63) | Model re-evaluated using the same cut-of as training model. | Isoleucine | 60.7 ^b^ | 70 | 52 | |
|  |  |  |  | Aspartic acid | 70.5 ^b^ | 70 | 71 | |
|  |  |  |  | Glutamate | 71.3 ^b^ | 78 | 65 | |
|  |  |  |  | Asparagine | 55.7 ^b^ | 61 | 51 | |
| Tristán, A. [2023] | Serum | CRC (n=57) vs. controls (n=26) | ROC analyses, no validation described for individual amino acids. | Alanine | 0.710 |  |  | |
|  |  |  |  | Glutamate | 0.770 |  |  | |
|  |  |  |  | Glutamine | 0.810 |  |  | |
|  |  |  |  | Isoleucine | 0.750 |  |  | |
|  |  |  |  | Valine | 0.710 |  |  | |
| Uchiyama, K. [2017] | Serum | CRC (n=56) vs. controls (n=60) | ROC analyses, no validation described for individual amino acids. | Histidine | 0.740 | 63 | 82 | |
| Zhu, j. [2014] | Serum | CRC (n=66) vs. controls (n=92) | ROC analyses, no validation described for individual amino acids. | Arginine | 0.611 (0.518-0.704) | 59 | 69 | |
|  |  |  |  | Aspartic acid | 0.649 (0.559-0.739) | 44 | 86 | |
|  |  |  |  | Glutamate | 0.660 (0.574-0.746) | 61 | 71 | |
|  |  |  |  | Glutamine | 0.652 (0.566-0.739) | 55 | 71 | |
|  |  |  |  | Histidine | 0.719 (0.640-0.789) | 92 | 47 | |
|  |  |  |  | Lysine | 0.680 (0.595-0.764) | 53 | 79 | |
|  |  |  |  | Methionine | 0.680 (0.596-0.764) | 67 | 65 | |
|  |  |  |  | Proline | 0.611 (0.521-0.702) | 42 | 79 | |
| Miyagi, y. [2011] | Plasma | CRC (n=199) vs. controls (n=995) | ROC analyses, no validation described for individual amino acids. ^a^ | Threonine | 0.377(0.330-0.419) |  |  | |
|  |  |  |  | Serine | 0.598 (0.559-0.647) |  |  | |
|  |  |  |  | Asparagine | 0.447 (0.402-0.495) |  |  | |
|  |  |  |  | Glutamine | 0.558 (0.559-0.647) |  |  | |
|  |  |  |  | Proline | 0.575 (0.530-0.615) |  |  | |
|  |  |  |  | Glycine | 0.577 (0.536-0.619) |  |  | |
|  |  |  |  | Alanine | 0.551 (0.501-0.594) |  |  | |
|  |  |  |  | Valine | 0.337 (0.294-0.383) |  |  | |
|  |  |  |  | Methionine | 0.416 (0.366-0.459) |  |  | |
|  |  |  |  | Isoleucine | 0.599 (0.554-0.643) |  |  | |
|  |  |  |  | Leucine | 0.472 (0.434-0.522) |  |  | |
|  |  |  |  | Tyrosine | 0.354 (0.314-0.399) |  |  | |
|  |  |  |  | Phenylalanine | 0.488 (0.422-0.537) |  |  | |
|  |  |  |  | Histidine | 0.283 (0.245-0.328) |  |  | |
|  |  |  |  | Tryptophan | 0.311 (0.263-0.359) |  |  | |
|  |  |  |  | Lysine | 0.500 (0.455-0.548) |  |  | |
|  |  |  |  | Arginine | 0.399 (0.353-0.448) |  |  | |
| Nishiumi, S. [2017] | Plasma | CRC (n=282) vs. controls (n=291) | ROC analyses, no validation described for individual amino acids. | Leucine | 0.593 | 49 | 66 | |
|  |  |  |  | Proline | 0.582 | 55 | 62 | |
|  |  |  |  | Cysteine | 0.649 | 79 | 46 | |
|  |  |  |  | Lysine | 0.622 | 39 | 82 | |
|  |  |  |  | Valine | 0.595 | 47 | 69 | |
|  |  |  |  | Tryptophan | 0.657 | 45 | 81 | |
| Lin, Y [2019] | Tissue | CRC (n=50) vs. controls (n=50) | ROC analyses, no validation described for individual amino acids. | Glutamate | 0.670 (0.523–0.797) |  |  | |
|  |  |  |  | Alanine | 0.749 (0.606–0.861) |  |  | |
|  |  |  |  | Glycine | 0.632 (0.484–0.764) |  |  | |
|  |  |  |  | Glutamine | 0.711 (0.583–0.817) |  |  | |
| Ning, W. [2017] | Tissue | CRC (n=5) vs. controls (n=5) | Markers selected on training set (CRC n=15, controls n=15). | Alanine | 0.753 | 80 | 75 | |
|  |  |  |  | Aspartic acid | 0.893 | 90 | 80 | |
| Su, H. [2025] | Saliva | CRC (n=35) vs. controls (n=36) | ROC analyses, no validation described for individual amino acids. | Methionine | 0.765(0.644-0.887) |  |  | |
| Kim, E. [2019] | Urine | CRC + AA (n=30) vs. controls (n=52) | Markers selected on training set (CRC+AA n=62 vs. controls n=104). | Alanine | 0.783 (0.714-0.839) |  |  | |

Abbreviations: AUC area under the curve, CI confidence interval, CRC colorectal cancer, MEV microbial extracellular vesicles, ROC receiver operator characteristic.

^b^ Reported values are accuracy.
